# Protometabolically Generated NADH Mediates Material Properties of Aqueous Dispersions to Coacervate Microdroplets

**DOI:** 10.1021/acs.biomac.5c00349

**Published:** 2025-07-18

**Authors:** Rudrarup Bose, Daniele Rossetto, Anju Tomar, Sanguen Lee, Sheref S. Mansy, T.-Y. Dora Tang

**Affiliations:** † 28271 Max Planck Institute of Molecular Cell Biology and Genetics, Pfotenhauerstrasse 108, 01307 Dresden, Germany; ‡ DiCIBIO, University of Trento, Via Sommarive 9, TN 38123 Povo, Italy; § Department of Pharmacy, Pharmaceutical Materials and Processing, Saarland University, 9379 PharmaScienceHub (PSH), Campus C4.1, 66123 Saarbrücken, Germany; ∥ Helmholtz Institute of Pharmaceutical Research Saarland (HIPS)-Helmholtz Centre for Infection Research (HZI), Campus E8.1, 66123 Saarbrücken, Germany

## Abstract

Macromolecular assembly between biomolecules dictates the material state of chemically complex aqueous dispersions such as the cytoplasm. The formation of protein precipitates, fibers, or liquid droplets have been associated with metabolic regulation and disease. However, the effect of metabolic flux on the material properties of aqueous dispersions remains underexplored. Here, we use the protometabolic reduction of NAD^+^ to NADH by pyruvate to study the effect of NADH production on the phase separation properties of polyarginine. We show that reduction of NAD^+^ in the presence of polyarginine can tune the material properties of the dispersion between precipitates, homogeneous solution, and liquid droplets depending on the buffer concentration. In situ droplet formation results in 2–3 times higher reaction rate and NADH yield, compared to homogeneous solution. Our study provides a setting for coupling protometabolism to active protocell environments in the absence of enzymes and sheds light on the self-regulation of metabolic flux on mediating biomolecular phase separation.

## Graphical abstract

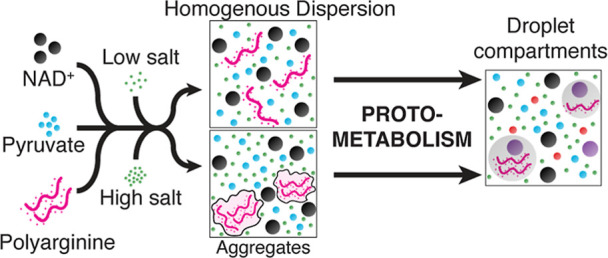

## 1 Introduction

A hallmark of life is its ability to sustain an out-of-equilibrium state that exploits metabolism and compartmentalization.^1,2^ Although metabolic reactions produce the essential biomolecules and energy within an organism, it is widely accepted that autopoietic organization via compartmentalization is a necessary condition for the emergence of life.^3^ The biological cell functions as a dynamic network of metabolic reactions that produces molecules that are used by the same network or processed by interconnected metabolic cycles.^4^ Further to this, extant cells are composed of active materials that exist in a wide variety of kinetic and thermodynamic material states. These can include liquid and gel-like droplets or precipitates and fibers where the phase behavior is dependent on the strength and type of intermolecular interactions; molecular composition and concentration. It has been shown that these material states can be regulated by metabolites,^5,6^ underlying biochemical reactions or external stress.^7^ Metabolic enzymes have been shown to form a variety of states.^8–11^ For example, liver phosphofructokinase (PFK) forms fibers; in yeast cells, glycolytic enzymes and RNA phase separate by liquid–liquid phase separation (LLPS) under stress conditions to regulate glycolysis.^12^ The reconfiguration of metabolic enzymes into compartments or fibers could contain, regulate, and protect metabolism.^10^ Despite the evidence that protein assemblies with different material states can affect biochemical reactions and cellular physiology,^13^ there have been a limited number of experiments which have focused on understanding the effect of small molecules, such as salt ions^14,15^ or metabolites, on modulating the material state of chemically complex aqueous dispersions. To the best of our knowledge, there are no studies which couple protometabolism with the material state of aqueous dispersions. Further consideration of the concentration and flux of metabolites in mediating the material properties of the cytoplasm can provide new insights into the coupling of dynamic biochemical processes with active material states in the cytoplasm. This provides the foundation for gaining deeper insights into the synergistic relationship between compartmentalization and metabolism in origin of life scenarios and extant biology.

To address this, we sought to use the intrinsic phase behavior of polyelectrolytes to explore the role of NADH, a key metabolite, for mediating the phases formed by polypeptide complexation. Polyelectrolytes can exhibit complex phase behavior,^16^ forming precipitates or droplets under specific conditions. Polyelectrolyte solutions will precipitate above a critical salt concentration (H-type) or in the case of strongly charged polymer solutions, small concentrations of multivalent ions can induce precipitation in a polymer concentration-dependent manner (L-type).^17^ The properties of the ions will also tune the ability for a polyelectrolyte to precipitate,^18^ that can be affected by the ionic radii, shape, and type of ion. In general, salt-induced precipitation is correlated with the Hofmeister series NH^4+^ > CS^+^ > Rb^+^ > K^+^ > Na^+^ > Ca^2+^ > Mg^2+^. In addition, the propensity of a polyelectrolyte to precipitate or form droplets could be dependent on the osmotic strength of the ion and the properties of the polyelectrolyte, such as its intrinsic p*K*_a_. For instance, the interactions between the side chains and ions in solution are modulated by the protonation states of the side chains, which vary with pH according to their respective p*K*_a_ values.

Coacervation between two oppositely charged polyelectrolytes provide the thermodynamic driving force for membrane-free compartmentalization^19,20^ . They are considered relevant models for simulating crowded biological reaction mediums like the cytoplasm,^21^ as well as providing plausible routes to prebiotic compartmentalization.^22^ Coacervate dispersions have been shown to exert selection pressures on encapsulated reactions by the partitioning of molecules,^23^ by promoting^24,25^ and suppressing reactions^23^ as well as by shifting reaction equilibria^26^ in enzyme-catalyzed reactions that can lead to shape mediation in peptide–DNA coacervates.^27^ Nonenzymatic reactions involving small molecules such as the condensation of imine are shown to have higher rates and equilibrium constants in coacervates compared to bulk solution.^28,29^ In this case, this was attributed to an upconcentration of reactants in the coacervate droplets.

It has recently been shown that the presence of small biological molecules such as ATP which have aromatic and charged functional groups can mediate interactions between proteins to reduce their propensity for aggregation.^5,6,30^ To test the ability of metabolites such as NADH to mediate phase behavior within aqueous dispersions of polypeptides, we used a minimal test tube system that is prebiotically relevant. This comprised the reduction of NAD^+^ to NADH by the α-ketoacid pyruvate^31,32^ in the presence of polyarginine in sodium bicarbonate buffer solution (Figure 1a). Previous studies have discussed the prebiotic relevance of this redox reaction to produce NADH in situ in a nonenzymatic manner. Using this system for our studies has the additional advantage of removing any additional effect of enzymes on the phase properties of the system that allows direct observation of NADH on the phase properties of the dispersion. Polyarginine was chosen as the polyelectrolyte due to its propensity to form precipitates at high concentrations of NaHCO_3_ (Figure 1b) via π–π stacking of the amphiphilic guanidium groups and reduced chemical complexity that are typically used for protocell models. Alkaline bicarbonate solutions were chosen for these experiments to mimic a plausible geochemical environment of carbonate-rich lakes on prebiotic earth.^33^ As polyarginine can precipitate under specific salt conditions (Figure 1b) and forms liquid droplets with NADH,^34,35^ the minimal system of bicarbonate buffer, polyarginine, and protometabolic production of NADH is the ideal system to investigate the coupling between protometabolism and primitive compartmentalization. Our results show that in situ formation of NADH can mediate the material properties of polyarginine from a precipitate phase or homogeneous phase to liquid droplets. Further, the tendency to form liquid droplets will increase the production of NADH compared to that of homogeneous solutions in the absence of polyelectrolytes. Our results demonstrate a remarkable diversity in active phase behavior in simple molecular systems. Further, our results represent an early form of primitive self-regulation in protocell-like systems in the absence of enzymes and provide a simplified framework for considering the coupling of metabolism with biomolecular phase separation in extant biology.

**Figure 1:**
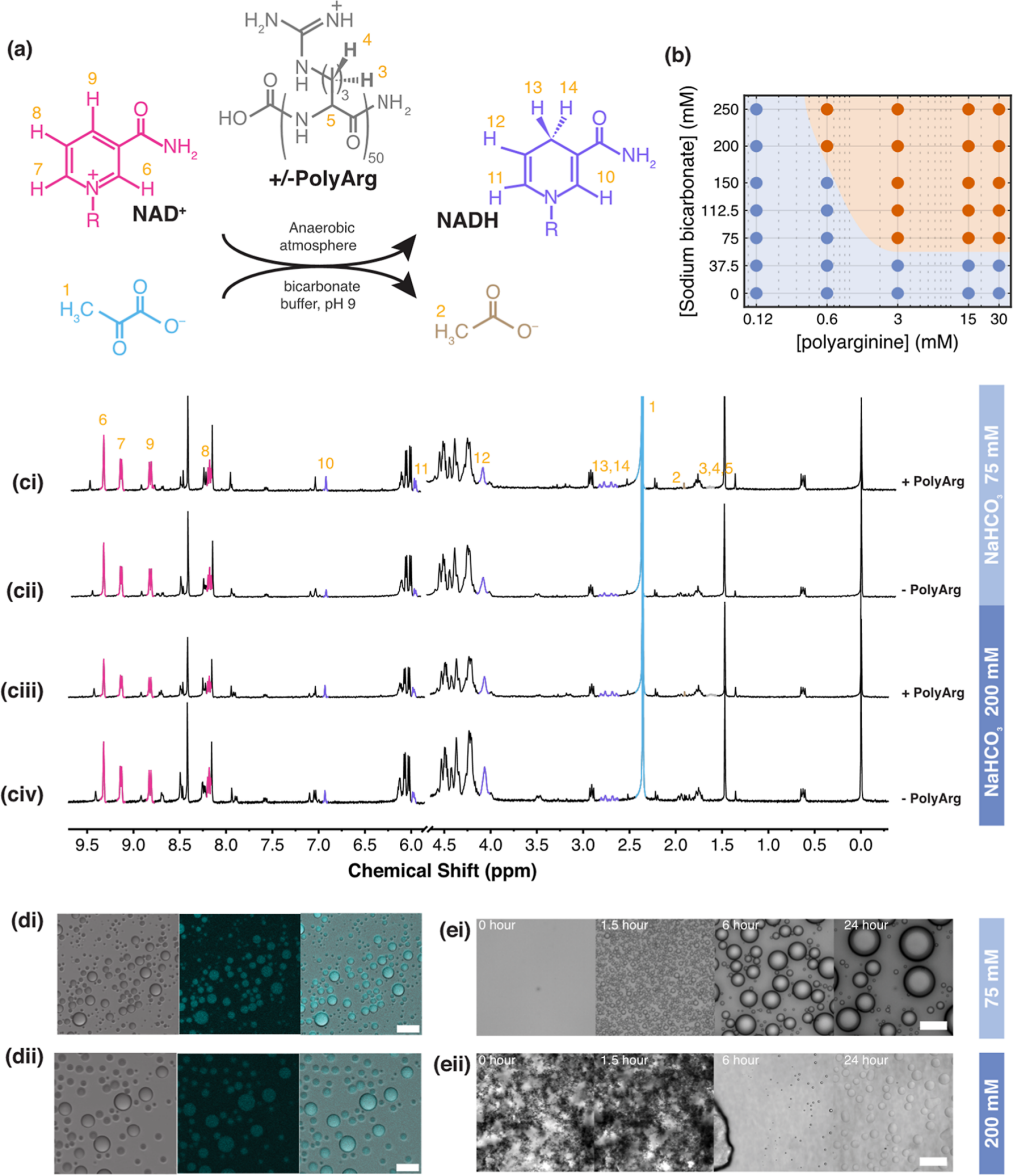
(a) Schematic of reaction, chemical structures, and protons identifiable by ^1^H NMR. (b) Phase diagram with semi-log plot of sodium bicarbonate and polyarginine (50 mer concentration). (c) ^1^H NMR spectra of reaction mixtures containing 15 mM NAD^+^ 30 mM pyruvate in either 75 mM sodium bicarbonate with (ci) 15 mM of polyarginine or (cii) in the absence of polyarginine or 200 mM sodium bicarbonate (ciii) 15 mM of polyarginine or (civ) in the absence of polyarginine. (d) Fluorescence microscopy imaging of coacervate droplets. Bright-field (left), fluorescence (center), and merged (right) images of the reaction with (di) 75 mM and (dii) 200 mM sodium bicarbonate buffer, showing droplets formed after 6 h from the start of the reaction (scale bar = 20 μm). Fluorescence images were acquired by illuminating with a light source of 355 nm and detecting emission between 449 and 506 nm. (e) Time-lapse bright-field images of the reaction with polyarginine at (ei) 75 mM and (eii) 200 mM sodium bicarbonate buffer show a transition to a droplet state.

## 2 Experimental Section

### 2.1 Materials

β-Nicotinamide adenine dinucleotide disodium salt (NAD^+^), β-nicotinamide adenine dinucleotide, reduced disodium salt (NADH), l-arginine monohydrochloride (arginine), l-lysine (lysine), NAD^+^/NADH quantitation kit, sodium pyruvate, and toluene were purchased from Sigma-Aldrich USA. Hydrochloric acid (HCl) and sodium hydroxide (NaOH) were purchased from Merck Millipore USA. Poly-l-arginine hydrochloride (polyarginine) and poly-l-lysine hydrochloride (polylysine) were purchased from Alamanda Polymers USA. 2-[Methoxy­(polyethyleneoxy)­propyl]­trimethoxysilane, 6–9 PEG units (PEG-silane) from ABCR, guanidine thiocyanate (GuSCN) from PanReac AppliChem Germany, sodium trimethylsilylpropanesulfonate (DSS) from Tokyo Chemical Industry Japan, sodium bicarbonate (NaHCO_3_) from Merck Germany, Hellmanex III from Hellma, and deuterated water (D_2_O) from Deutero GMBH Germany were purchased.

### 2.2 Preparation of Reaction Mixtures

In all experiments, reaction mixtures were prepared to a final concentration of 15 mM NAD^+^ and 30 mM sodium pyruvate with either 75 or 200 mM NaHCO_3_ buffer at pH 9 with and without 15 mM polyarginine. In addition, experiments were undertaken with 15 mM NAD^+^, 30 mM sodium pyruvate, and 75 mM NaHCO_3_ with either 15 or 150 mM of monomer arginine.

Milli-Q water and 500 mM NaHCO_3_ at pH 9.0 (adjusted with 7.5 M sodium hydroxide) were degassed using standard protocols. Solutions were placed in a Schlenk line and frozen on a mixture of acetone and dry ice. Oxygen was removed by a vacuum pump followed by thawing on warm water. To ensure that the maximum amount of oxygen had been removed, the procedure of freezing, vacuum, and thawing was repeated 5 times. The solutions were then stored under nitrogen flux for up to a week. At the time of the experiments, solutions were transferred from the Schlenk line into the glovebox in a Teflon capped vial, preconditioned with nitrogen flux.

Stock solutions (A–D) (Table 1) were prepared in a glovebox with a nitrogen flow and oxygen levels below 0.1% (measured using Okolab LEO hand-held meter) to ensure anaerobic conditions. NAD^+^, sodium pyruvate, polyarginine, and arginine solids were weighed into microcentrifuge tubes and transferred into the glove chamber along with degassed Milli-Q water and 500 mM sodium bicarbonate buffer (pH 9.0).

**Table 1:** List of Stock Solutions for the Reaction Mixture

solution	chemical mixture	aqueous solution
A	25 mM polyarginine or monomer arginine	Milli-Q water
B	250 mM polyarginine or monomer arginine	Milli-Q water
C	75 mM pyruvate, 37.5 mM NAD^+^	187.5 mM sodium bicarbonate buffer (pH 9)
D	75 mM pyruvate, 37.5 mM NAD^+^	500 mM sodium bicarbonate buffer (pH 9)

To prepare 100 μL of reaction mixture with 75 mM NaHCO_3_ buffer, 40 μL of solution C was added to 60 μL of solution A/solution B/or degassed water for a reaction mixture containing 15 mM (solution A) or 150 mM (solution B) of polyarginine or arginine or a reaction mixture free from peptides. To prepare reaction mixtures containing 200 mM NaHCO_3_, 40 μL of solution D was added to 60 μL of solution A, B, or degassed water to give a final concentration of 15 mM (solution A) or 150 mM (solution B) of polypeptide or amino acid or a reaction mixture free from polypeptide or amino acids.

### 2.3 Characterization of Reaction by NMR

Samples were prepared, as described previously, under anaerobic conditions. To produce the 600 μL of sample required for NMR experiments, the volumes were scaled up. For experiments undertaken in the absence of poly amino acids, 500 mM NaHCO_3_ buffer was prepared with 10% D_2_O water and 1 mM of sodium trimethylsilylpropanesulfonate (DSS) and degassed as previously described. For reactions that contained (poly) arginine, the reaction mixture was prepared as previously described. At specified time points, the sample was diluted with 10 mM DSS prepared in D_2_O to prepare a final reaction sample containing 10% of D_2_O and 1 mM DSS.

Samples were loaded into an NMR TUBE (Boroeco-5-7, Deutero) and inserted into a BRUKER NanoBay 400 MHz NMR spectrometer. ^1^H NMR spectra were obtained with water suppression using the Bruker’s noesygppr1d pulse program with 16 scans (fid size: 32768). In summary, the following settings were used: spectral width: 15.9794 ppm/6393.862 Hz, acquisition time: 2.5624576 s, fid resolution: 0.390250 Hz, filter width: 4032 kHz, transmitter frequency offset: 4.7 ppm/1880.61 Hz, receiver gain: 32, dwell time: 78.2 μs, DSP firmware filter: rectangle, digitization mode: baseopt, digitizer resolution: 22, homodecoupling duty cycle: 20%, oversampling during homodecoupling: 1, maximum variation of a delay: 5%, first step for PL switching: −6 dB, step width for PL switching: 0.1, MAS rotation: 4200 Hz, probe temperature: 298 K, temperature on channel: 300 K, gradient temperature: 300 K, number of wobble steps: 1024. The NMR spectra were analyzed by using Mestrelab Mnova software. The spectrum was adjusted using DSS as the reference, δ 0.00 (s, 9H). The ^1^H NMR spectra was compared against the assignable hydrogens in the ^1^H NMR spectrum of pure NADH, NAD^+^, pyruvate, acetate, and polyarginine, i.e., NADH, (β-nicotinamide adenine dinucleotide reduced disodium salt): ^1^H NMR (400 MHz, H_2_O with 10% D_2_O): δ 2.72 (dd, *J* = 14.3, 7.9 Hz, 2H), 5.97 (d, *J* = 8.2 Hz, 1H), 6.93 (s, 1H). NAD^+^, (β-nicotinamide adenine dinucleotide disodium salt), ^1^H NMR (400 MHz, H_2_O with 10% D_2_O): δ 8.18 (t, *J* = 7.2, 7.2 Hz, 1H), 8.82 (d, *J* = 8.1 Hz, 1H), 9.13 (d, *J* = 6.3 Hz, 1H), 9.32 (s, 1H). Pyruvate, (O=C–CH_3_)–C­(O^–^): ^1^H NMR (400 MHz, H_2_O with 10% D_2_O): δ 1.47 and 2.36 (s, 3H). Acetate, (O=C)–(CH_3_): ^1^H NMR (400 MHz, H_2_O with 10% D_2_O): δ 1.91 (s, 3H). Polyarginine, (−NH–CH­(CNH­(NH_2_))–(CH_2_)_5_–NH–C­(O)−)_*n*_: ^1^H NMR (400 MHz, D_2_O): δ 1.97–1.55 (br).

### 2.4 Imaging of In Situ Droplet Formation

Time-resolved widefield and confocal bright-field and fluorescence microscopy was used to image the reaction mixture. To ensure strict anaerobic conditions, 100 μL of the sample was loaded into 18-well chamber slide (Ibidi, Germany) in a glovebox (Bel-Art Techni-Dome 360° Glove Chamber) that was adhered to a pegylated coverslip. The 18-well slide was then sealed with an adhesive clear microplate sealing sheet (Thermo Scientific, USA). The slide was further sealed with high-vacuum grease (Dow, USA) (Figure S1). The microscope slide was loaded onto a Zeiss Axiovert 200 M wide-field microscope within a Pecon Incubator HF 2000 and imaged at 1% oxygen level, controlled through Okolab CO_2_–O_2_ Unit-BL [0-10,1-18]. Images were taken every 15 min with an Andor Zyla PLUS monochrome sCMOS camera with 6.5 μm dexel size and 20×/0.4 LD A-Plan, air, Ph2, Zeiss objective (product ID: 421051-9910-000). Multiwell imaging was conducted on 6 wells with 15 ms exposure, gain = 3 and digitizer = 200 MHz. Z-stacks were obtained with step size of 3 μm over a range of 80 μm.

To image NADH^36^ inside the droplets, fluorescence microscopy was conducted using Zeiss LSM 880 airy inverted confocal microscope with Plan-Apochromat 20×/0.8 M27. For illumination of NADH, a 355 nm laser source, with laser power 1.2 μW (0.375 μW/cm^2^), master gain 700, and digital gain 1, was used in conjunction with a MBS355 beam splitter. The detection filter was set between 449 and 506 nm. Bright-field images were obtained using the transmission detection module (T-PMT) when the sample was illuminated with a 561 nm laser source, with laser power 6.7 μW (2.075 μW/cm^2^), master gain 250, and digital gain 1, in conjunction with MBS488/561 beam splitter. For imaging 30.5 μm pinhole size along with 7 μs scan time, averaging of 4 and bit depth of 8 were used.

### 2.5 Phase Diagram Characterization

To determine the phase behavior of NAD^+^ and NADH with polyamino acids, we characterized the phase diagrams of polyarginine (0.15, 1.5, 7.5, 15, and 30 mM) with NAD^+^ or NADH (0.15, 1.5, 7.5, 15, and 30 mM) in either 75 mM or 200 mM NaHCO_3_ buffer. To do this, the following stock solutions were prepared: 60 mM NAD^+^ in 150 mM NaHCO_3_ buffer (pH 9); 60 mM NAD^+^ in 400 mM NaHCO_3_ buffer (pH 9); 60 mM NADH in 150 mM NaHCO_3_ buffer (pH 9); 60 mM NADH in 400 mM NaHCO_3_ buffer (pH 9); and 60 mM polyarginine in Milli-Q (∼pH 7). In all instances, the pH of NaHCO_3_ was adjusted to pH 9 using NaOH unless otherwise stated.

In addition, the phase behavior of arginine (15 mM) was determined with NADH (0.5, 1, 5 mM) in 75 and 200 mM NaHCO_3_. To do this, stock solutions of 30 mM arginine were prepared in sodium bicarbonate buffer (75 mM and 200 mM, pH 9). All stock solutions were stored at −20 °C until further use. The monomer concentration of the polypeptide was reported as the concentration of the polypeptide solution.

To prepare samples for phase behavior characterization, stock solutions were diluted in their respective buffers or Milli-Q water to enable a 1:1 volume mixing between polyarginine or arginine with NAD^+^ or NADH to achieve the final concentration. For example, to prepare samples containing 0.15 mM polyarginine, 0.15 mM NADH, and 75 mM NaHCO_3_, 60 mM polyarginine in Milli-Q water was diluted to 0.30 mM polyarginine with Milli-Q water. Solutions containing 60 mM NADH in 150 mM NaHCO_3_ were diluted to 0.30 mM NADH with 150 mM NaHCO_3_. The solution of polypeptide or peptide was mixed with NADH or NAD^+^ at equal volumes to produce 20 μL of polyarginine/NADH/NaHCO_3_ solutions.

Widefield microscopy imaging was used to determine the phase state of polypeptide with NAD^+^/NADH in either 75 or 200 mM NaHCO_3_. To do this, 10 μL of the sample was loaded into a capillary slide formed from strips of parafilm, sandwiched between a glass slide and a pegylated coverslip.^37^ Coverslips were pegylated to prevent droplet wetting on the surface. In brief, coverslips were first cleaned with Hellmanex III (Hellma) and then with distilled water by sonicating for 5 min in each solution. The wash steps were repeated 3 times, and the coverslips were then fully submerged into a well-mixed solution of 100 mL of toluene, 460 mg of PEG silane, and 160 μL of 36% hydrochloric acid. The coverslips were left for 18 h with constant stirring and covered to prevent evaporation. The coverslips were then rinsed with pure toluene, then 2 times with pure ethanol and distilled water, respectively. Finally, coverslips were dried with nitrogen and stored under vacuum until use.

Once the sample was loaded into the capillary slide, the samples were imaged with a Zeiss Axiovert 200 M wide-field microscope equipped with an Andor Zyla 4.2 (VSC-02370) camera with 6.5 μm dexel size and a Zeiss 100×/1.3 oil Plan-Neofluar Ph3M27 objective. To determine the phase of the sample, images were analyzed by eye to determine whether droplets or precipitates were present. To image samples that did not contain precipitates or droplets, 0.2 μL of 0.02% solution of micrometer-sized carboxylate beads (Polybead, Polysciences Europe GmbH, product ID: 08226-15), prepared in Milli-Q water, was added to 10 μL of the mixture and imaged using bright-field microscopy to locate the surface of the coverslip.

### 2.6 Determination of the Fraction of NADH Produced

To quantify NADH formation as a function of time, we used a commercially available dual enzyme assay (NAD^+^/NADH quantitation kit, Sigma-Aldrich, USA) which measures the relative amount of NADH compared to NAD^+^ and NADH (Figure S2).

$${\left(NADH\right)}_{rel}=NADH/\left({NAD}^{+}+NADH\right)$$

To obtain the relative amount of NADH in the reaction mixture, the reaction mixture was prepared as previously described and incubated under anaerobic conditions. At a specified time point, 4 M guanidine thiocyanate (GuSCN) was added to the reaction mixtures at 1:1 volume to dissolve the coacervate droplets at a final concentration of 2 M GuSCN.

To obtain the amount of NADH in the solution, half of the reaction mixture containing 2 M GuSCN was diluted 1000-fold in NAD^+^ extraction buffer (supplied in the NAD^+^/NADH quantitation kit) and incubated for 30 min at 60 °C to degrade all NAD^+^. The NAD^+^/NADH quantification assay was then performed as described by the manufacturer’s instructions.

To obtain the total amount of NAD^+^ and NADH, the other half of the reaction mixture containing 2 M GuSCN was diluted 10,000-fold in the NAD^+^ extraction buffer and then assayed with the NAD^+^/NADH quantification kit according to the manufacturer’s instructions.

For both reaction mixtures, 25 μL of the diluted reaction mixture was loaded into a 96-well microplate (F-bottom, clear, Greiner Bio-One, Austria) along with 50 μL of the enzyme mix. After 5 min of incubation, 5 μL of substrate II was added to achieve a total volume of 80 μL. After the color had developed, 5 μL of stop solution was added as described by the manufacturer’s instructions. With each experiment, a control experiment was performed with the known concentrations of NADH (0, 0.4, 0.8, 1.2, 1.6, 2 μM) as provided by the manufacturer. 25 μL of the NADH standards was incubated with 50 μL of enzyme mixture in a well plate reader alongside the reaction samples. Once the absorbance had reached a value between 1.0 and 1.5, the reaction mixture was quenched with the 5 μL of stop solution from the assay kit and the samples were measured by absorbance using a TECAN Spark 20M well plate reader at room temperature (Figure S3a).

The calibration curve was used to determine the absolute quantity of NADH and (NAD^+^ + NADH) from each of the separated reaction mixtures. The concentrations of (NADH) and (NAD^+^ + NADH) were scaled relative to the dilution factors. Three repeats were undertaken for each time point and each sample.

(NADH)_rel_ was plotted as a function of time, and the values of (NADH)_rel_ for the first 8 h were fit to a straight-line without intercept, using MATLAB, to obtain the initial rate of the reaction (Figure S3b). The standard deviation was reported as the error associated with the slope. This was calculated by dividing the range of the 95% confidence interval by 3.92 (since the 95% confidence interval of a prediction is equal to the predicted value ±1.96 times the standard deviation).

## 3 Results and Discussion

We first tested the electron transfer reaction between NAD^+^ (15 mM) and pyruvate (30 mM) in sodium bicarbonate buffer, at pH 9 as previously described,^31^ in the presence of polyarginine (15 mM) and at two different buffer conditions (75 mM and 200 mM). After 24 h, all reactions were centrifuged, and the supernatant was removed and loaded into an NMR tube with 10% D_2_O for analysis by NMR spectroscopy (Figures 1 and S4–S11). Comparison of the reaction mixtures, in the absence and presence of polyarginine (50 mer) and at 75 and 200 mM sodium bicarbonate buffer confirmed the production of NADH and acetate with polyarginine and at 75 mM (low) and 200 mM (high) carbonate concentrations (Figure 1c). To determine the phase state of the reaction mixture with polyarginine, we used optical microscopy to image the reaction after 6 h. Confocal microscopy images showed the presence of microdroplets that were fluorescent upon UV excitation, λ_exc_ = 340 ± 30 nm and λ_emi_ at 450 ± 50 nm. We observed fluorescence emission commensurate with NADH within the microdroplet (Figure 1d). In this case, NADH could form the structural component (scaffold) of the coacervate or act as the client by partitioning into the droplet. Given that polyarginine will precipitate above 75 mM of sodium bicarbonate (Figure 1b), we used time-resolved widefield optical microscopy to detect any phase change in the dispersion during the course of the reaction. At 200 mM sodium bicarbonate, the reaction mixture in the presence of polyarginine showed precipitates which transitioned to droplets within 24 h (Figure 1e). In comparison, at lower sodium bicarbonate concentration (75 mM), there was a homogeneous dispersion at the start of the reaction which then transitioned to droplets within 2 h of the reaction (Figure 1e). To determine whether the phase transition was a thermodynamically or kinetically driven process, we added NADH (30 mM) to a dispersion of polyarginine precipitates (15 mM) in sodium bicarbonate buffer (200 mM). Optical microscopy images showed a transition from precipitate to droplets appearing within 5 s of NADH addition (see Supporting Information 2.3: Supporting Note 1). Given that in the presence of the protometabolic reaction, droplets are observed on the time scale of minutes; these experiments indicate that protometabolic-driven phase transitions are limited by the in situ formation of NADH. Taken together, the results confirm that nonenzymatic electron transfer reactions can take place in the presence of polyarginine and that the generation of NADH could be responsible for a change in phase properties within the reaction mixture.

To further confirm that production of NADH was responsible for the observed phase transitions, we mapped the thermodynamic phase diagrams of polyarginine (0–30 mM) with NADH (0–30 mM) in 75 and 200 mM sodium bicarbonate (Figures 2 and S14–S16). The results confirm that NADH can form droplets with polyarginine in both of these conditions. Interestingly, the phase diagrams show a precipitate region toward the bottom right of the phase diagram (low NADH concentration and high polyarginine concentration) (Figure 2a). This phase region is larger at 200 mM sodium bicarbonate compared to 75 mM bicarbonate. The phase diagrams show that increasing the NADH concentration will lead to a transition between a precipitate phase and a droplet phase. It is interesting to note that while the phase diagrams show a precipitate at 75 mM bicarbonate and 15 mM polyarginine, no precipitates were observed in the reaction mixture that contained pyruvate immediately after mixing, indicating that pyruvate will affect the phase behavior of the overall system (Figure 1ei).

**Figure 2:**
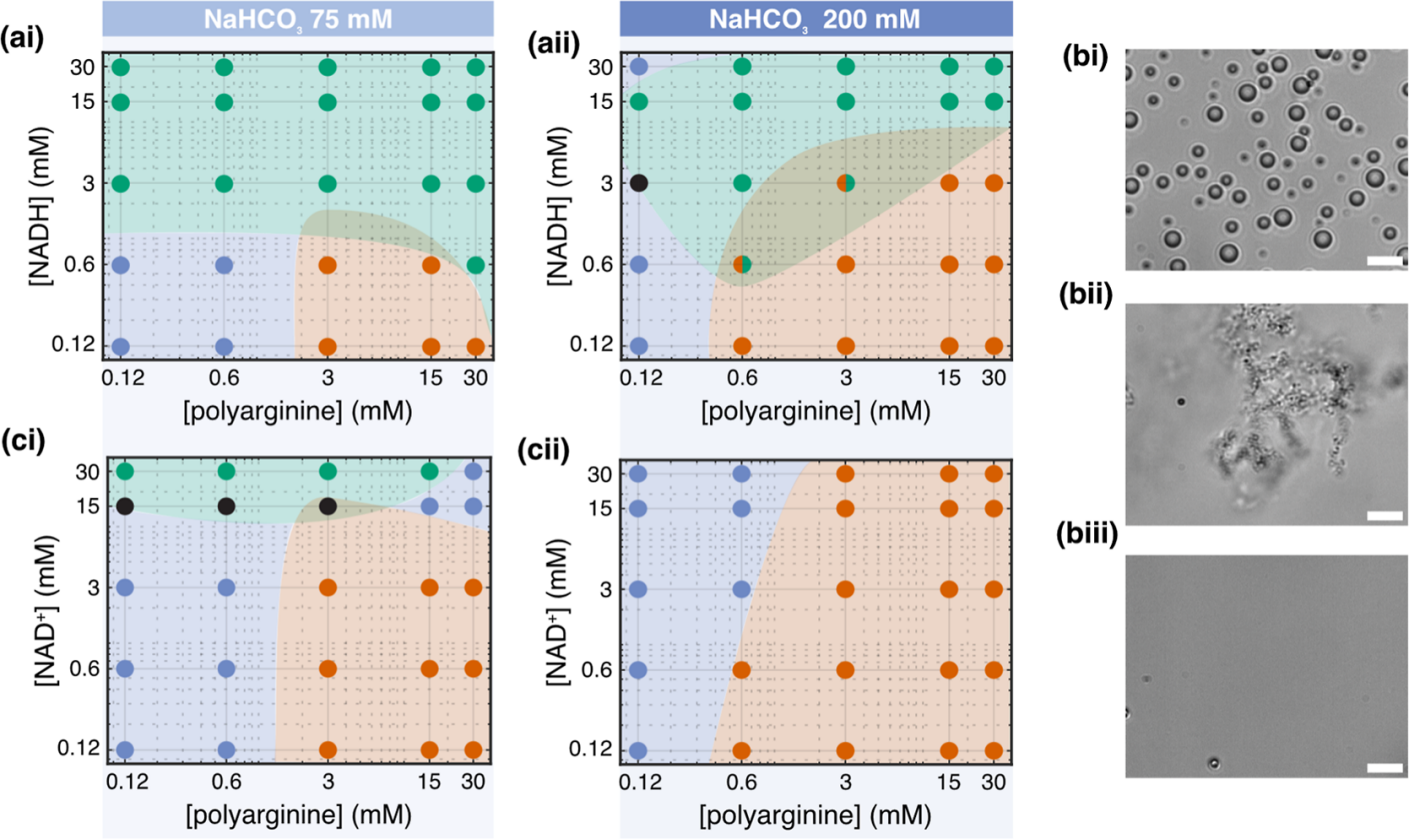
Phase behavior of polyarginine and NAD^+^/NADH (a) shows the phase diagram of 50-mer polyarginine with NADH in 75 mM (ai) and 200 mM (aii) sodium bicarbonate, respectively, in a log–log plot. (b) Representative images of droplets (15 mM polyarginine, 3 mM NADH in 75 mM sodium bicarbonate) (i), precipitate (15 mM polyarginine, 15 mM NAD^+^ in 200 mM sodium bicarbonate) (ii), and a homogeneous phase (15 mM polyarginine, 15 mM NAD^+^ in 75 mM sodium bicarbonate) (iii). Scale bar = 5 μm. (c) Phase behavior of 50-mer polyarginine with NAD^+^ in 75 and 200 mM sodium bicarbonate, respectively, in a log–log plot. Blue, red, and green markers represent dissolved solutions, precipitates, and droplets. Conditions for which we were unable to determine whether droplet or precipitate were present with certainty are marked black.

Further, the phase diagrams show that mixtures of polyarginine, NADH, and buffer can lead to three phase regions (precipitates, droplets, and homogeneous phases (Figure 2b)). In order to decipher the stabilizing factors for this diverse phase behavior, we further mapped the phase diagrams of polyarginine in sodium bicarbonate buffer (75 and 200 mM) with NAD^+^ (Figure 2c). At 200 mM of sodium bicarbonate, we observe two phase regions (a homogeneous phase and a precipitate phase), and at lower buffer conditions (75 mM), we observe three phases: a homogeneous phase; a precipitate phase; and a droplet phase. The droplet phase is stabilized at high NAD^+^ concentrations. Comparing phase diagrams with NAD^+^ and NADH shows that NADH increases the droplet phase region even under high buffer conditions.

In summary, the phase diagrams show that increased buffer concentration leads to stabilization of precipitates that could be driven by hydrophobic interactions.^38^ On the other hand, NAD^+^/NADH with polyarginine favors droplet formation with NADH having a stronger effect than NAD^+^. As NADH is the reduced form of NAD^+^, NADH is more negatively charged, suggesting that increased electrostatic interactions stabilize the polyarginine droplets. Taken together, this could suggest that in multicomponent systems, different interactions stabilize different phases. In this instance, hydrophobic interactions at high salt and polypeptide concentrations drive precipitate formation, while electrostatic interactions lead to droplet formation. The balance in the interactions based on concentration could mediate material states within aqueous dispersions.

Given that we observe different materials properties, we next sought to determine the effect of the material states on the kinetics of NADH production. To do this, we measured the fraction of NADH (of the total NAD^+^ and NADH) as a function of time. This was done using a commercially available biochemical assay that was undertaken on the NAD^+^/NADH reaction (see Materials and Methods and Figures S2, S3, and S18) at 75 and 200 mM sodium bicarbonate in the absence and presence of polyarginine (15 mM). Our results show that after 24 h, the presence of polyarginine leads to 2–3 times greater relative NADH (NADH_rel_ ∼ 0.3) compared to dispersions without polyarginine (NADH_rel_ ∼ 0.1) (Figure 3a,c). In these latter samples, there was no significant difference in the reaction profiles for the two buffer conditions. However, comparison between 75 and 200 mM sodium bicarbonate with polyarginine showed that more NADH was produced at the lower concentration of sodium bicarbonate (NADH_rel_ = 0.34 ± 0.04) compared to the higher buffer conditions (NADH_rel_ = 0.25 ± 0.01). Comparison of the initial rates showed that the absence of polyarginine gave a lower initial rate (0.0073 ± 0.0008 h^–1^ with 75 mM sodium bicarbonate and 0.0071 ± 0.0006 h^–1^ in 200 mM sodium bicarbonate) compared to the reaction mixture with polyarginine (0.0160 ± 0.002 h^–1^ in 75 mM sodium bicarbonate and 0.0120 ± 0.0011 h^–1^ with 200 mM sodium bicarbonate) (Figures 3b and S17).

**Figure 3:**
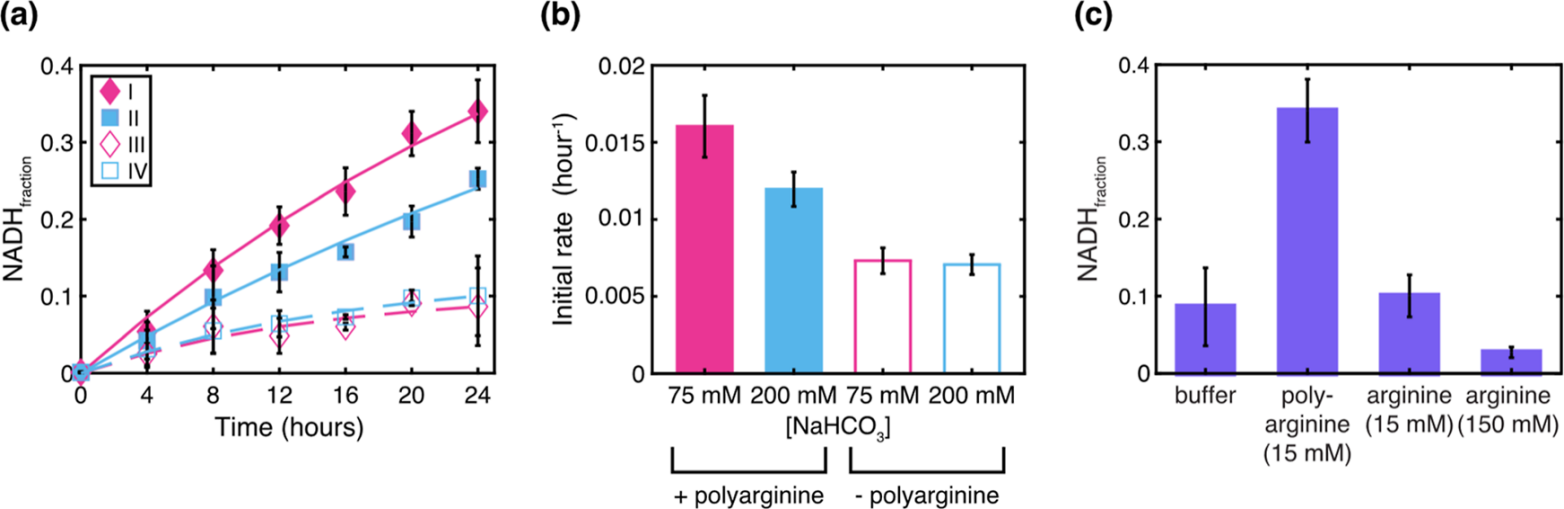
Quantification of the NADH production using commercially available biochemical assay. (a) Comparison of the NADH_rel_ ([NAD_reduced_^+^] ÷ [NAD_total_^+^]), measured at different time points up to 24 h from the start of the protometabolic reaction, in the presence of polyarginine at 75 mM sodium bicarbonate buffer (I), in the presence of polyarginine at 200 mM sodium bicarbonate buffer (II), in the absence of polyarginine at 75 mM sodium bicarbonate buffer (III), and in the absence of polyarginine at 200 mM sodium bicarbonate buffer (IV). (b) Comparison of the initial rate at the start of the reaction calculated by fitting a straight-line, without intercept, to each of the data sets until 8 h. (c) Fraction of NADH produced after 24 h from the start of the protometabolic reaction in the presence of 75 mM sodium bicarbonate alone and with added polycations, 15 mM 50-mer polyarginine, 15 mM arginine, and 150 mM arginine.

The observed differences in the fraction of NADH after 24 h and the initial rate of NADH production could be attributed to increased charge from polyarginine rather than the phase properties. To test this, we measured the fraction of NADH at 24 h, with monomeric arginine at 15 and 150 mM to simulate a charged environment in the absence of a physical droplet. In both cases, we observed that the NADH fraction after 24 h remained lower in comparison to reactions undertaken with 15 mM polyarginine that formed coacervate droplets. These results show that the increase in NADH production in the presence of polypeptide is attributed to the environment, which is created by the material state. Moreover, the results show that in situ formation of NADH modulates the material state of the dispersion from a single phase to droplets and from precipitates to droplets under different buffer conditions.

## 4 Conclusion

In conclusion, we used a minimal system that comprises a nonenzymatic, protometabolic electron transfer reaction with a polypeptide to test the ability for NADH to mediate different material states within an aqueous dispersion. With this simple system, we demonstrate that in situ production of NADH can drive a transition from a homogeneous solution to a dispersion with droplets and from dispersions of precipitates to droplets. We propose that molecular mixtures can exhibit a variety of different phases that are stabilized by dominating interactions, such as the hydrophobic effect (precipitates) and electrostatic interactions (liquid droplets), that are dependent on the chemical and physical conditions of the aqueous dispersions. While our studies have focused on polyarginine, it should be possible that other polyelectrolytes that have both hydrophobic and charged elements could exhibit both the propensity to form precipitates and droplets. Indeed, chitosan, an amphiphilic polyelectrolyte, will form precipitates at high salt concentrations which transition to droplets after the addition of NADH with phase properties comparable to polyarginine (see Supporting Information 2.5: Supporting Note 2, Figures S19 and S20). In addition, the ability for small molecules to induce a transition between precipitate phase to a droplet phase is not isolated to NADH but can be extended to other metabolically relevant molecules such as ATP, CoA, and FAD (see Supporting Information 2.6: Supporting Note 3, Figure S21). This suggests that the multiphase behavior of amphiphilic polyelectrolytes driven by metabolites could be a general phenomenon given the correct conditions.

Further to this, we show that heterogeneous environments (droplet phases) lead to an increase in the rate and amount of NADH compared to homogeneous dispersions, which we attribute to the spatial rather than the chemical environment. The ability for NADH to mediate material states within aqueous protein dispersions (see Supporting Information 2.7: Supporting Note 4 Figure S22) is important for considering how metabolism can actively tune and regulate the structural properties of the cytoplasm and how heterogeneous environments could regulate metabolism. Further, it would be pertinent to consider the consequences of an active prebiotic soup during the origin of life where carbonate-rich lakes could have provided a plausible scenario for the origins of life,^33^ and concentrations of bicarbonate and salts can change upon water evaporation in wet–dry cycling scenarios.^39,40^ These different scenarios coupled with prebiotic chemical reactions can impact the solubility of biologically relevant molecules, the chemical and material states, and the outcomes of prebiotic reactions. These insights underscore the impact of early metabolic activities on the emergence of protocellular systems, by solubilizing precipitated polymers and leading to the formation of localized environments conducive to chemical networks, even in salt-rich environments. This active prebiotic soup potentially mirrors the properties of cytoplasm in extant biology, containing various phases that can both mediate and be mediated by chemical or biochemical reactions.

## Supplementary Material



## Data Availability

All data is available at the following doi: https://doi.org/10.17617/3.QFXGQA.

## Notes

Methods; results and notes: NMR; bright-field microscopy images of polyarginine phase behavior; kinetically driven droplet formation; determination of fraction of NADH produced; multiphase behaviour in amphiphilic polyelectrolytes; effect of metabolites on polyarginine–carbonate precipitates; and effect of NADH on chicken egg white albumin (PDF)

## ⊥

Department of Synthetic Biology, University of Saarland, Campus Gebäude B2.2, 66123 Saarland, Germany

## Abbreviations

NAD^+^: nicotine adenine dinucleotide
PFK: 6-phosphofructokinase
RNA: ribonucleic acid
NMR: nuclear magnetic resonance
