# Supplementary material for: Protometabolically Generated NADH Mediates Material Properties of Aqueous Dispersions to Coacervate Microdroplets
Source: Biomacromolecules. 2025 Jul 18;27(1):112–20. doi: 10.1021/acs.biomac.5c00349 (PMC12801307; doi:10.1021/acs.biomac.5c00349)
Supplement: Supplementary file 1 [file bm5c00349_si_001.pdf]

Supporting Information

Protometabolically generated NADH mediates material properties of aqueous dispersions to coacervate microdroplets

Rudrarup Bose[a], Daniele Rossetto[a], Anju Tomar[b], Sanguen Lee[c,d], Sheref S. Mansy[b], T-Y Dora Tang[,a]*

[a]Max Planck Institute of Molecular Cell Biology and Genetics, Pfotenhauerstrasse 108, 01307 Dresden, Germany

[b]DiCIBIO, University of Trento, Via Sommarive 9, Povo, TN 38123, Italy

[c] Department of Pharmacy, Pharmaceutical Materials and Processing, Saarland University, PharmaScienceHub (PSH), Campus C4.1, 66123 Saarbrücken, Germany

[d] Helmholtz Institute of Pharmaceutical Research Saarland (HIPS)- Helmholtz Centre for Infection Research (HZI), Campus E8.1, 66123 Saarbrücken, Germany

Contents:

1. **Supplementary Materials and methods**

2. **Supplementary Results and notes**

   2.1.NMR

   2.2. Bright field microscopy images of polyarginine phase behaviour

   2.3. Supplementary Note 1: Droplet formation is kinetically driven

   2.4. Determination of fraction of NADH produced

   2.5. Supplementary Note 2: Multiphase behaviour of amphiphilic polyelectrolytes

   2.6. Supplementary Note 3: Effect of metabolites on polyarginine-carbonate precipitates

   2.7. Supplementary Note 4: Effect of NADH on chicken egg white albumin

1. **Supplementary Materials and methods**

Table S1. Detailed list of materials used.

**Chemical Name**	**Molecular Weight**	**Supplier**	**Product identifier**	**CAS number**
**2-[methoxy(polyethyleneoxy)propyltrimethoxysilane, 6-9 PEG units (PEG-silane)**	458.62 – 590.77 g mol^{-1}	ABCR	AB111226	65994-07-2
**β-nicotinamide adenine dinucleotide disodium salt (NAD^+)**	685.41 g mol^{-1}	Sigma Aldrich	N0632	20111-18-6
**β-nicotinamide adenine dinucleotide, reduced disodium salt (NADH)**	709.4 g mol^{-1}	Sigma Aldrich	N8129	606-68-8
**Adenosine Triphosphate disodium salt hydrate (ATP)**	561.1 g mol^{-1}	Sigma Aldrich	A3377-5G	34369-07-8

**Ammonium Sulfate**	132.1 g mol^{-1}	Grüssing Gmbh	101551000	7783-20-2
**Chitosan**	20-100 kDa	Heppe Medical Chitosan GmbH	23301	9012-76-4
**Coenzyme A (CoA) hydrate**	767.5 g mol^{-1}	Sigma Aldrich	C4282-100MG	85-61-0
**Deuterated Water (D_2O)**	20.03 g mol^{-1}	Deutero	00506	7789-20-0
**Flavin adenine dinucleotide disodium salt hydrate (FAD)**	829.5 g mol^{-1}	Sigma Aldrich	F6625-100MG	84366-81-4
**Guanidine thiocyanate (GuSCN)**	118.16 g mol^{-1}	PanReac AppliChem	A1107	593-84-0
**Hydrochloric acid (HCl)**	36.46 g mol^{-1}	Merck Millipore	1.00317	7647-01-0
**Hellmanex III**	N / A	Hellma	9-307-011-4-507	N / A

**L-arginine monohydrochloride (arginine)**	210.66 g mol^{-1}	Sigma Aldrich	A5131	1119-34-2
**L-lysine (lysine)**	146.19 g mol^{-1}	Sigma Aldrich	L5501	200-294-2
**NAD^+/NADH quantitation kit**	N / A	Sigma Aldrich	MAK037	N /A
**Piperazine-*N*,*N′*-bis(2-ethanesulfonic acid (PIPES)**	302.37 g mol^{-1}	Sigma Aldrich	P6757	5625-37-6
**Polyethylene glycol (PEG) 8000**	8000 kDa	G-Biosciences	RC-077	25322-68-3
**Poly-L-arginine hydrochloride (polyarginine)**	9.6 kDa (~ 50 mer)	Alamanda Polymers USA	PLR50	26982-20-7
**Poly-L-lysine hydrochloride (polylysine)**	8.2 kDa (~ 50 mer)	Alamanda Polymers USA	PLKC50	26124-78-7

**Poly(N-isopropylacrylamide) PNIPAM**	10000 M_n (average)	Sigma-Aldrich	724459-5G	23189-55-3
**Sodium Acetate**	82.03 g mol^{-1}	Sigma Aldrich	241245	127-09-3
**Sodium bicarbonate ($NaHCO_3$)**	84.01 g mol^{-1}	Merck Germany	1.06329	144-55-8
**Sodium hydroxide (NaOH)**	40.00 g mol^{-1}	Merck Millipore USA	1.06498	1310-73-2
**Sodium pyruvate**	110.04 g mol^{-1}	Sigma Aldrich	P2256	113-24-6
**Sodium trimethylsilylpropanesulfonate (DSS)**	218.32 g mol^{-1}	Tokyo Chemical Industry	T1638	2039-96-5
**Toluene**	92.14 g mol^{-1}	Sigma Aldrich	32249-M	108-88-3

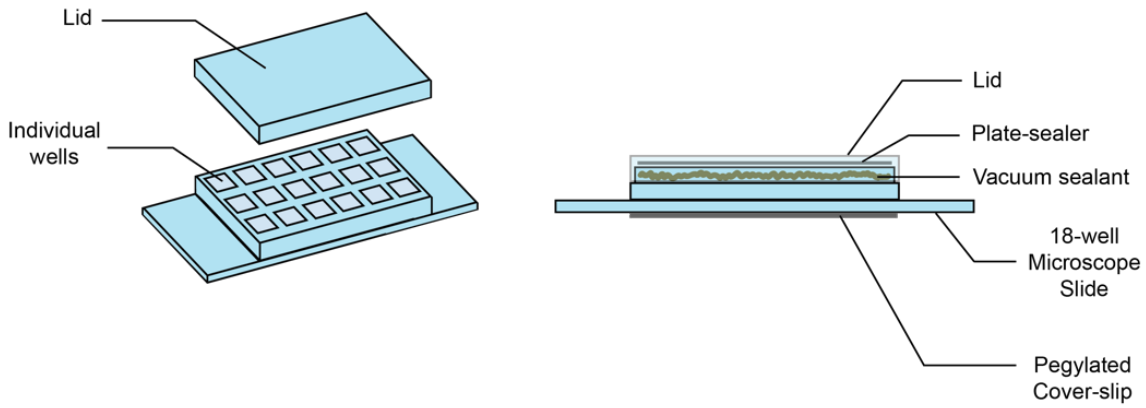

Figure S1. Microscope slide set-up for imaging experiments. Schematic representation of the 18-well bottomless slide, consisting of a sticky underside Left: top view. Right: side view

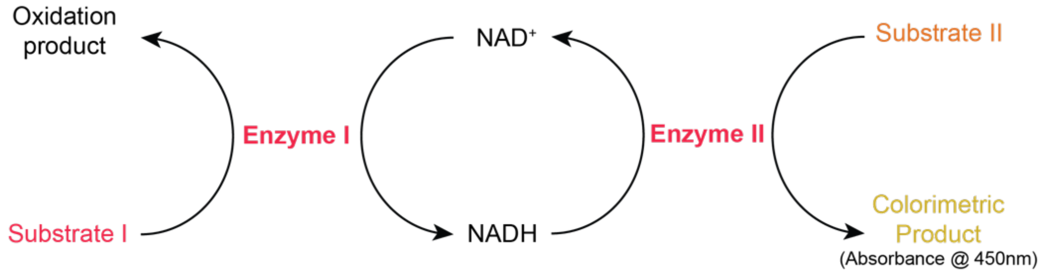

Figure S2. Schematic of commercially available NAD^+/NADH quantitation assay. The assay contains Solution 1 containing Enzymes I and II as well as the substrate I. This enzyme mix converts NAD^+ to NADH. The developer solution contains substrate II and a chromogenic substrate that converts all NADH to NAD^+, coupled with the conversion of the chromogenic substrate to a colorimetric product that absorbs at 450 nm.

(a)

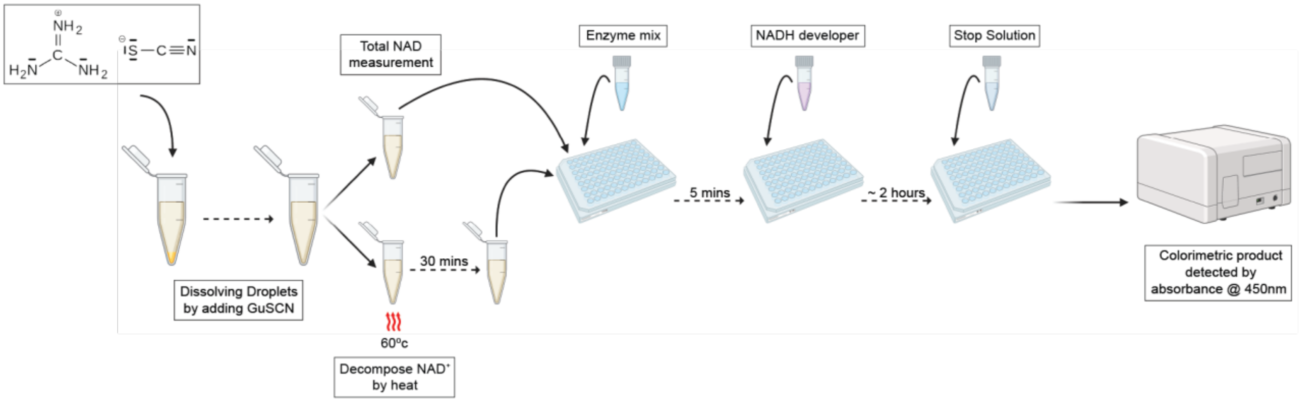

(b)

NADH Standard Solution

0 µM	0.4 µM	0.8 mM	1.2 µM	1.6 µM	2.0 µM
0.2284	0.5917	0.9322	1.3518	1.4303	1.6325
0.2263	0.8013	0.955	1.3826	1.4784	1.7202

Sample

NAD^+ + NADH	NADH
0.4056	0.2910
0.3794	0.3006

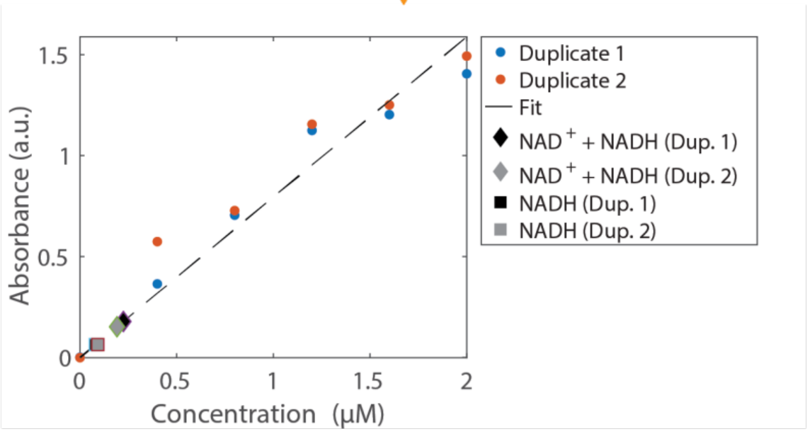

NAD^+ + NADH (mM)		NADH (mM)		$NADH_{rel}$	
Dup. 1	Dup. 2	Dup. 1	Dup. 2	Dup. 1	Dup. 2
4.4883	3.8286	0.1603	0.1844	0.0357	0.0482

Figure S3. Schematic describing the measurement of NADH using NAD^+/NADH quantitation assay. (a) describes the steps involved in performing the NAD^+/NADH quantitation assay. First, the samples were diluted 1:1 with 4 M guanidine thiocyanate (GuSCN), to dissolve coacervate droplets. The sample was diluted in the assay buffer, supplied by the manufacturer. The sample was further diluted 1000 times and then split into two parts. One part was heated for 30 minutes at 60 °C to degrade NAD^+, while the other part was diluted a further 10-fold and used directly in the assay. The former constitutes the sample NADH, while the latter sample NAD^+ + NADH. To perform the assay, the enzyme mix was added to both the samples and controls containing 0 μM, 0.4 μM, 0.8 μM, 1.2 μM, 1.6 μM and 2.0 μM of NADH standard, in duplicates. This was followed by the addition of NADH developer solution after an incubation period of 5 minutes. The absorbance of the controls at 450 nm was measured in a TECAN Spark 20M wellplate reader, until the maximum value of absorbance (i.e. of 2.0 μM NADH) reached 1.0 – 2.0. A stop solution, also supplied by the manufacturer, was then added to arrest the enzymatic activity and prevent the degradation of the colorimetric product. After the addition of the stop solution the samples and the controls were then measured for absorbance at 450 nm. (b) shows the detailed steps of the data analysis for a single sample, as an illustrative example. The absorbance values of the controls were plotted against the corresponding NADH concentration. This is then linearly fitted, using least squares' method, to the straight line Y = mX, where Y is the absorbance and X is the corresponding concentrations, to obtain the slope, m. Using the slope, the concentration of NADH and NAD^+ + NADH was obtained for each duplicate as concentration = (absorbance / slope) × dilution factor. The amount of NADH was reported as $NADH_{rel}$, such that for each duplicate $NADH_{rel}$ = NADH (mM) ÷ (mean of 2 duplicates of NAD^+ (mM) + NADH (mM)).

2. Supplementary Results

2.1. NMR

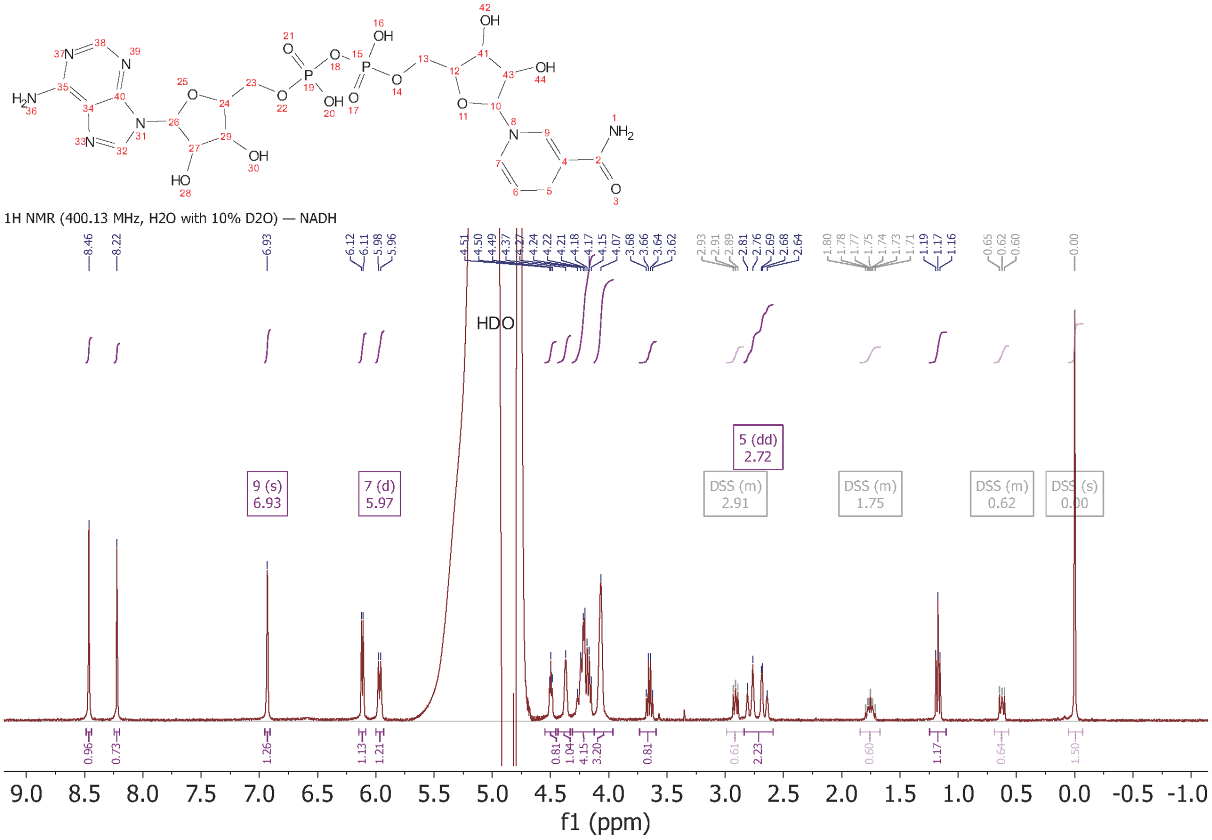

Figure S4. ^{1}H NMR spectra of NADH (400 MHz, H_2O with 10% D_2O) with DSS internal standard. The assignable hydrogens have been marked on the spectra. For reference the structure is provided; δ 2.72 (dd, J = 14.3, 7.9 Hz, 2H), 5.97 (d, J = 8.2 Hz, 1H), 6.93 (s, 1H).

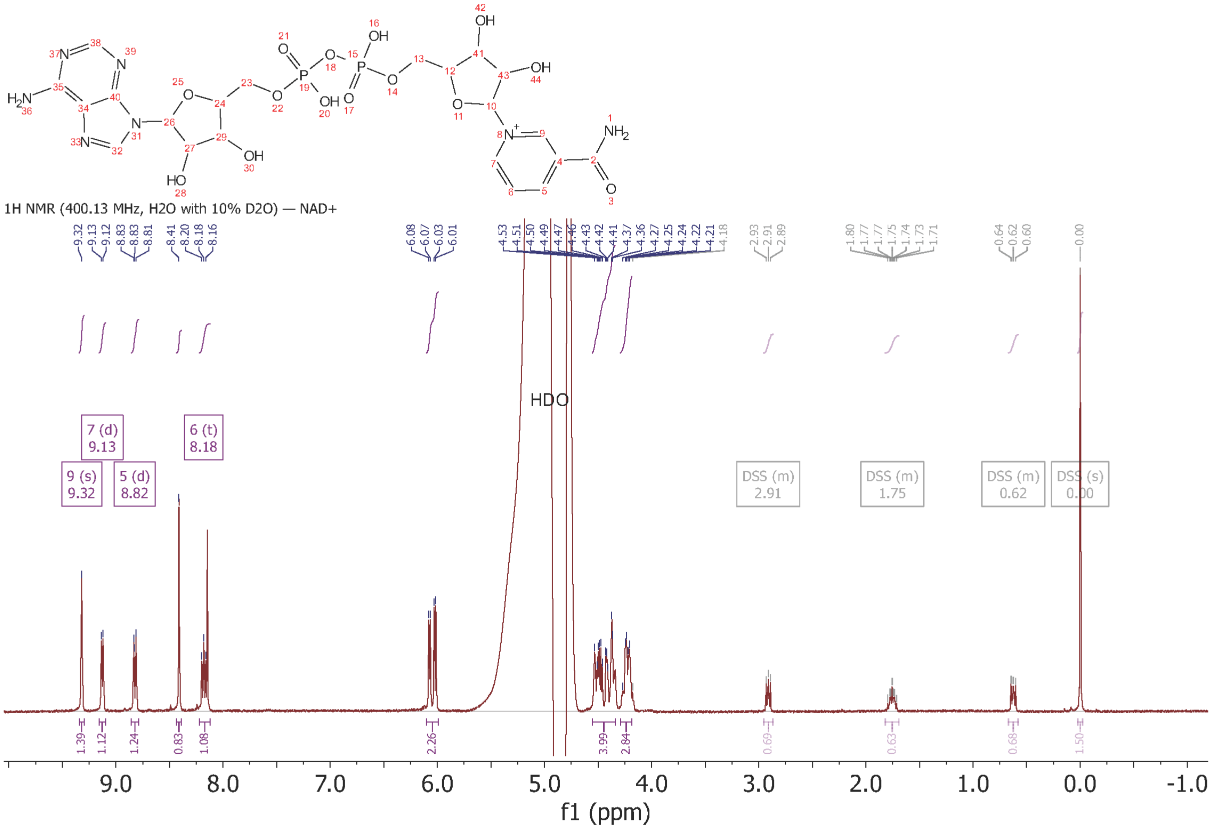

Figure S5. ^{1}H NMR spectra of NAD^{+} (400 MHz, H_2O with 10% D_2O) with DSS internal standard. The assignable hydrogens have been marked on the spectra. For reference the structure is provided; δ 8.18 (t, J = 7.2, 7.2 Hz, 1H), 8.82 (d, J = 8.1 Hz, 1H), 9.13 (d, J = 6.3 Hz, 1H), 9.32 (s, 1H).

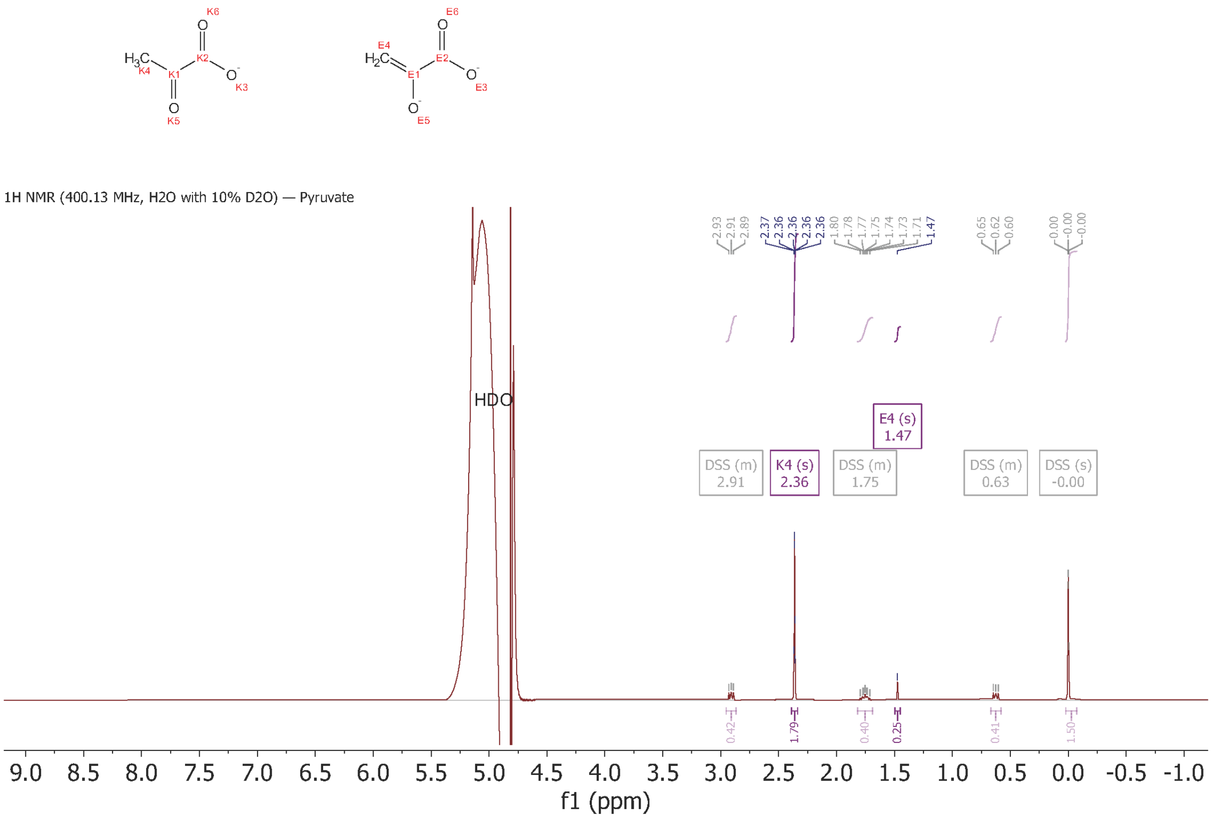

Figure S6. ^{1}H NMR spectra of pyruvate (400 MHz, H_2O with 10% D_2O) with DSS internal standard. The assignable hydrogens have been marked on the spectra. The structures are provided for reference; δ 1.47 & 2.36 (s, 3H).

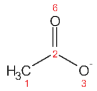

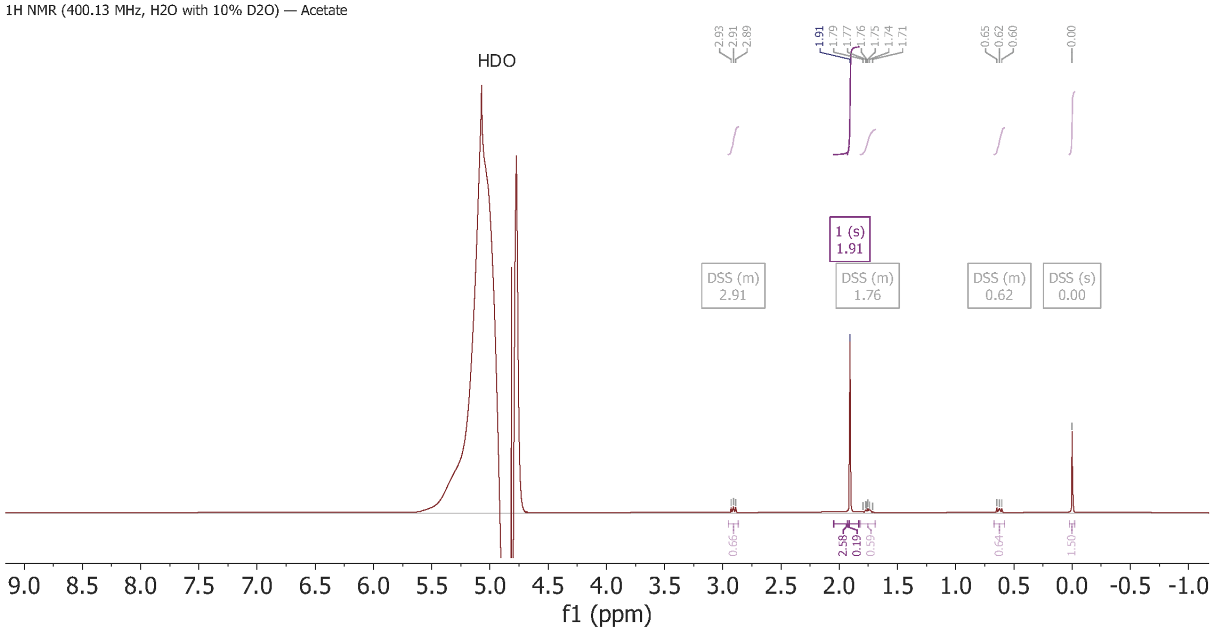

Figure S7. ^{1}H NMR spectra of Acetate (400 MHz, H_2O with 10% D_2O) with DSS internal standard. The assignable hydrogens have been marked on the spectra. The structure is provided for reference; δ 1.91 (s, 3H).

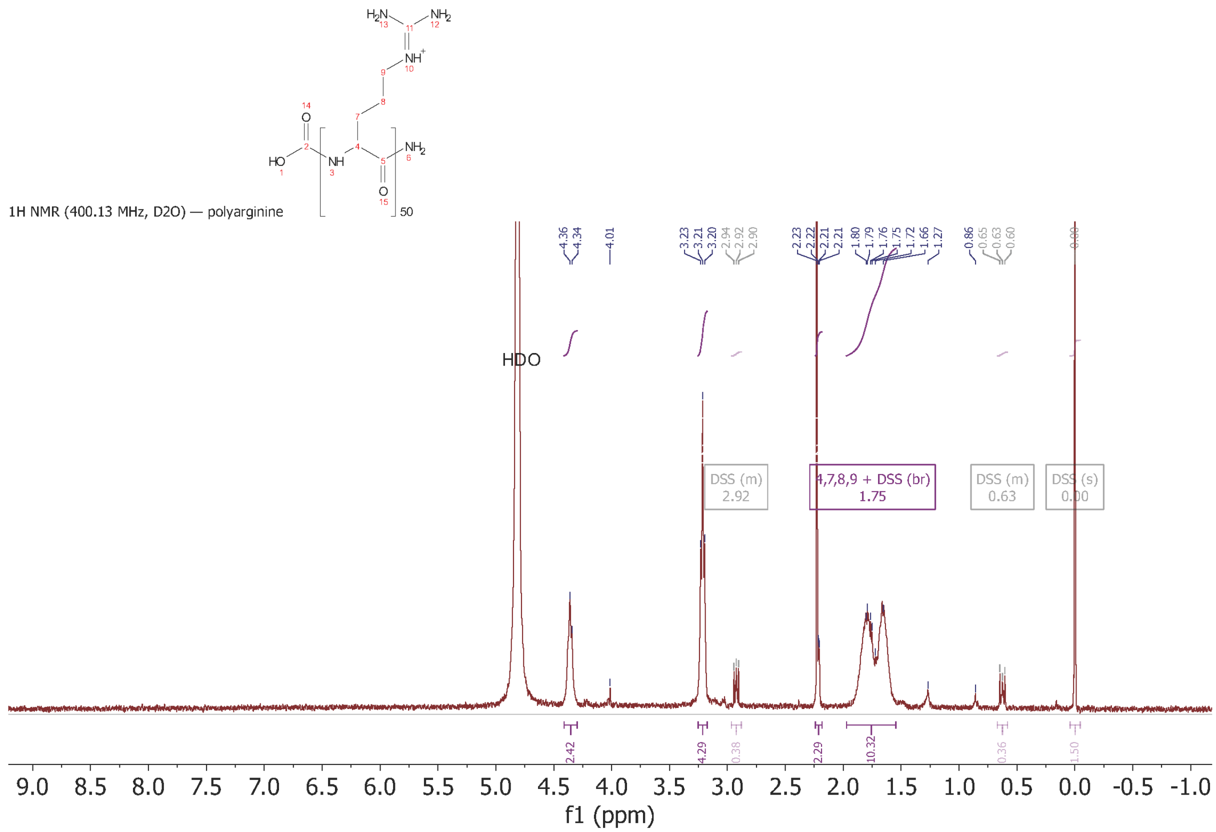

Figure S8. ^{1}H NMR spectra of polyarginine (400 MHz, D_2O) with DSS internal standard. The assignable hydrogens have been marked on the spectra. For reference the structure is provided; δ 1.97 – 1.55 (br).

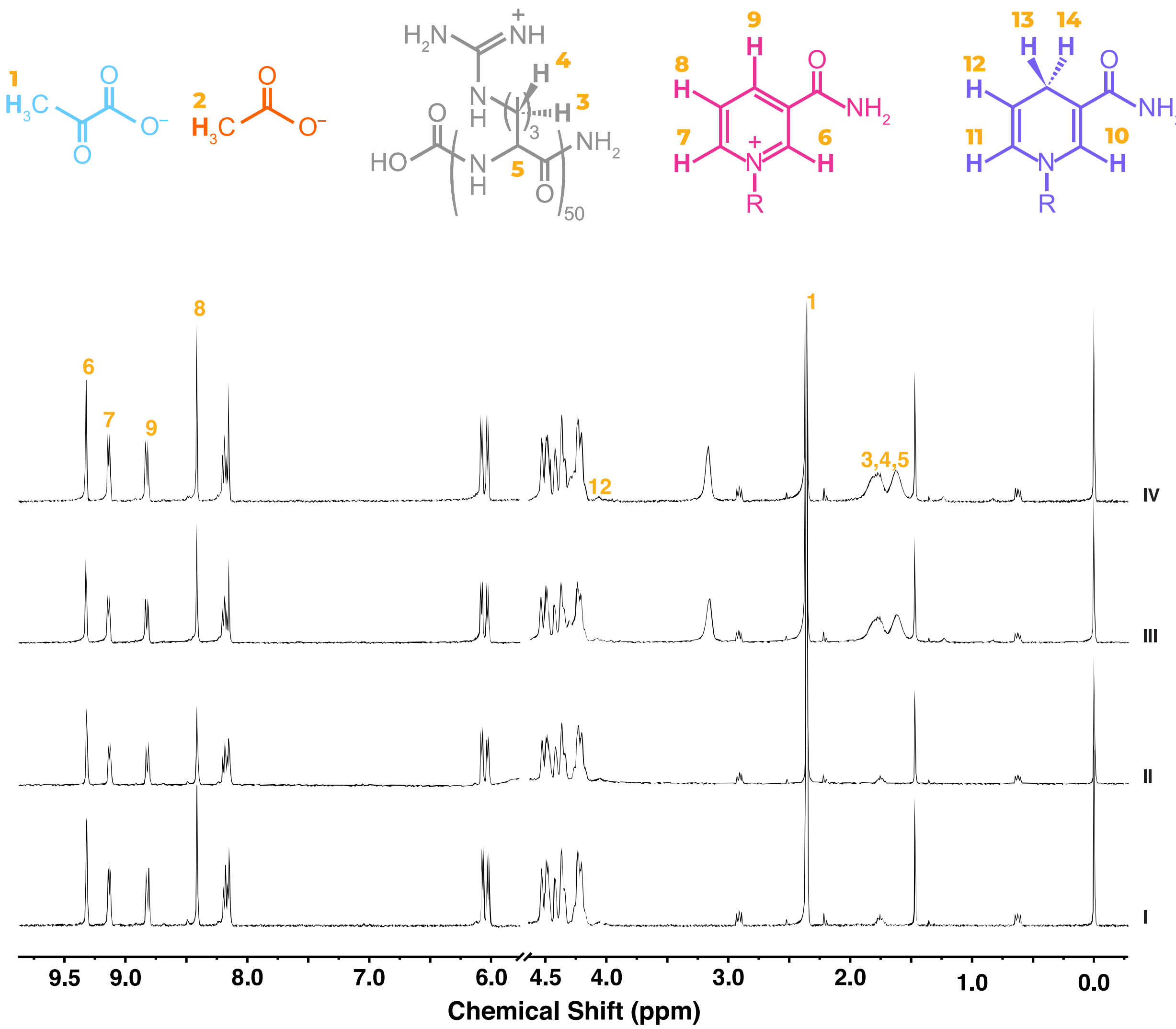

Figure S9. ^{1}H NMR spectra of reaction mixtures at the start of the reaction. I, II, III and IV are reaction mixtures without polyarginine in 75 mM $NaHCO_3$ buffer, without polyarginine in 200 mM $NaHCO_3$ buffer, with 15 mM polyarginine in 75 mM $NaHCO_3$ buffer and with 15 mM polyarginine in 200 mM $NaHCO_3$ buffer.

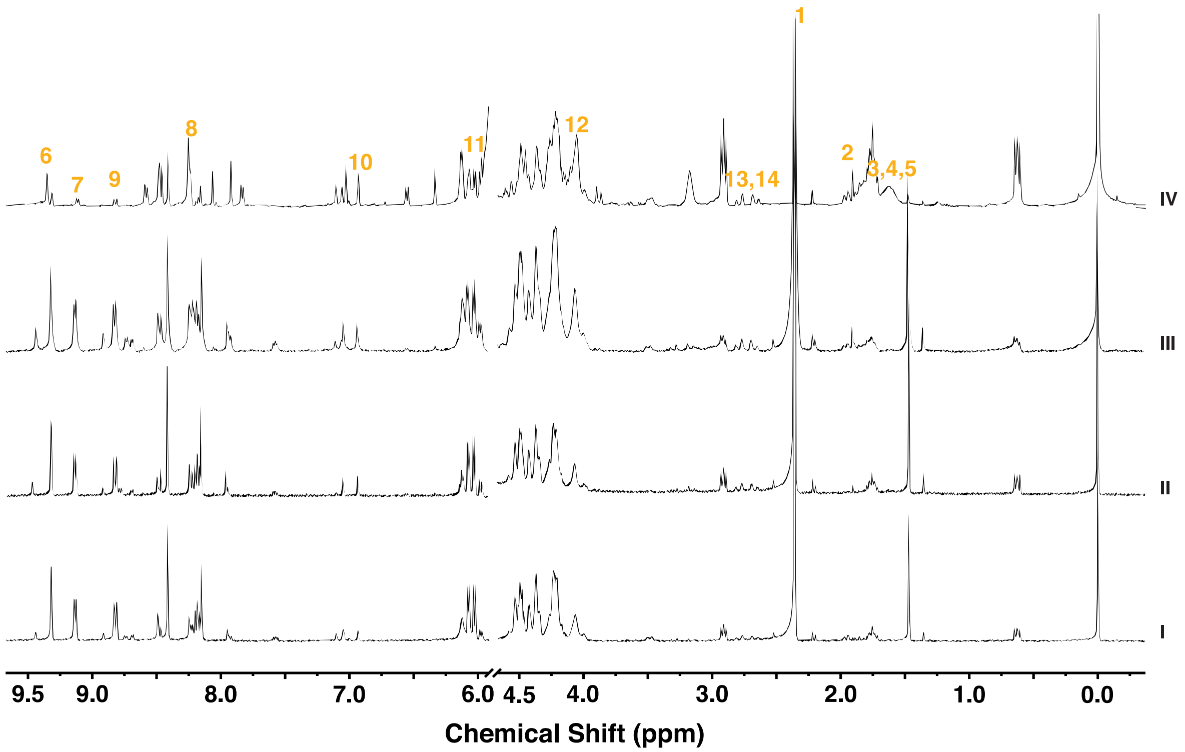

Figure S10. ^{1}H NMR spectra of reaction mixtures prepared in 75 mM $NaHCO_3$ buffer after 24 hours of the reaction. I and II are reaction mixtures without polyarginine and with 15 mM polyarginine. Reaction mixtures with 15 mM polyarginine were centrifuged to separate the dilute phase and the coacervate phase. III and IV show the ^{1}H-NMR spectra of the dilute and the coacervate phase redissolved in 75 mM $NaHCO_3$ buffer.

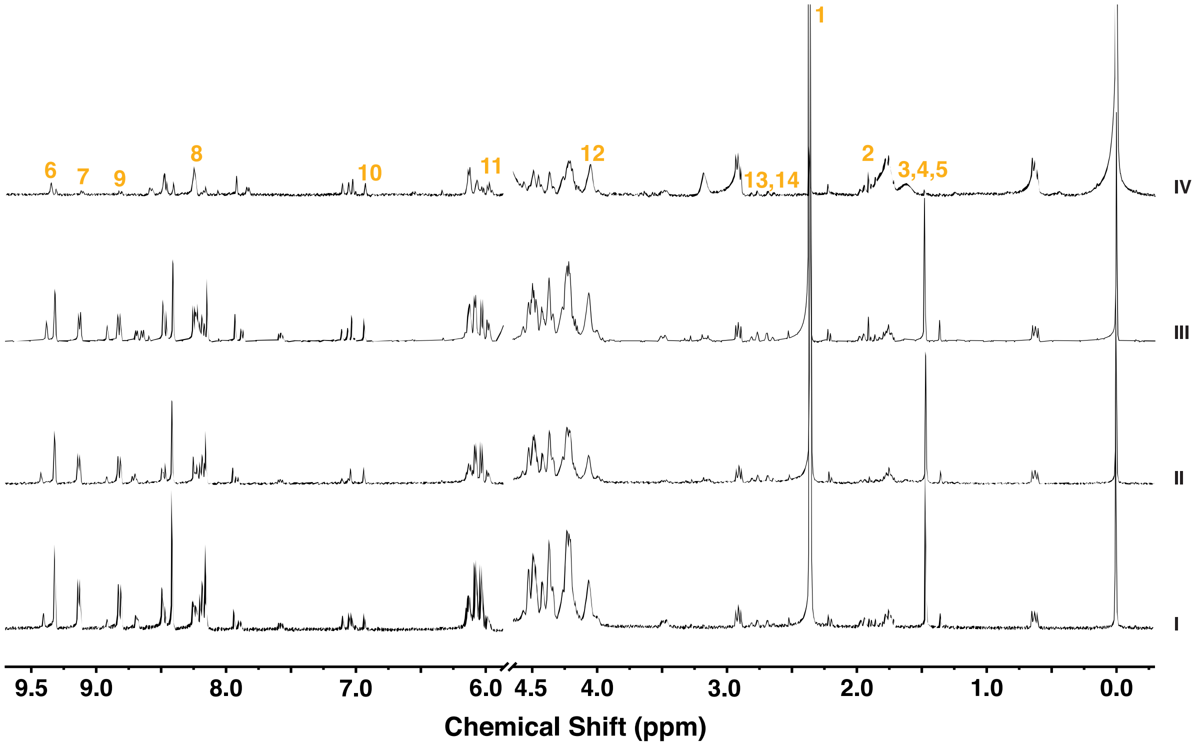

Figure S11. ^{1}H NMR spectra of reaction mixtures prepared in 200 mM $NaHCO_3$ buffer after 24 hours of the reaction. I and II are reaction mixtures without polyarginine and with 15 mM polyarginine. Reaction mixtures with 15 mM polyarginine were centrifuged to separate the dilute phase and the coacervate phase. III and IV the ^{1}H-NMR spectra of the dilute and the coacervate phase redissolved in 200 mM $NaHCO_3$ buffer.

2.2.Bright field microscopy images of polyarginine phase behaviour

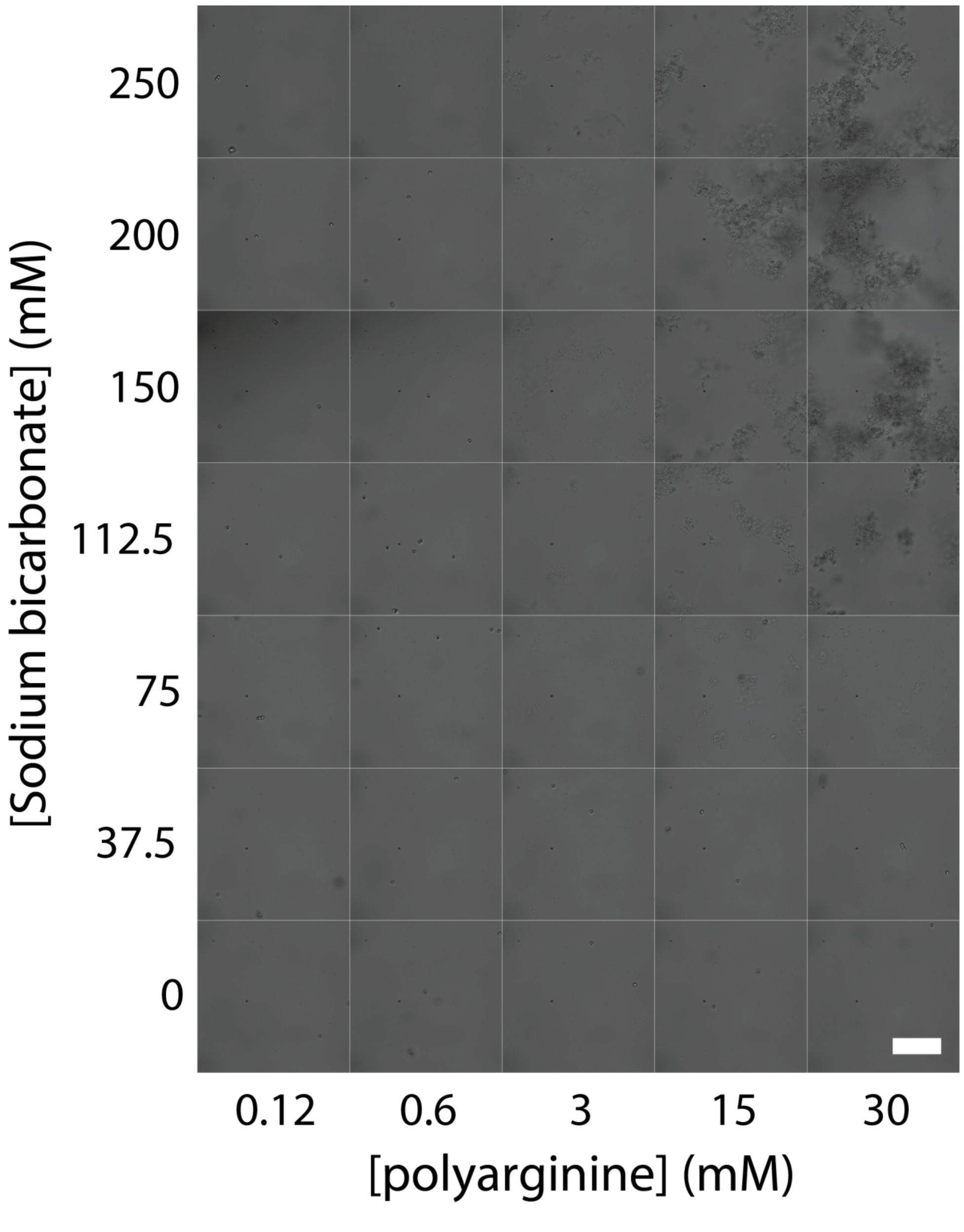

Figure S12. Bright field microscopy images of mixtures of sodium bicarbonate and 50-mer polyarginine showing their phase behaviour, comprised of either dissolved or a precipitated state. To aid visualisation, carboxylate microspheres were added to samples that did not contain precipitates. Scale bar : 20 µm.

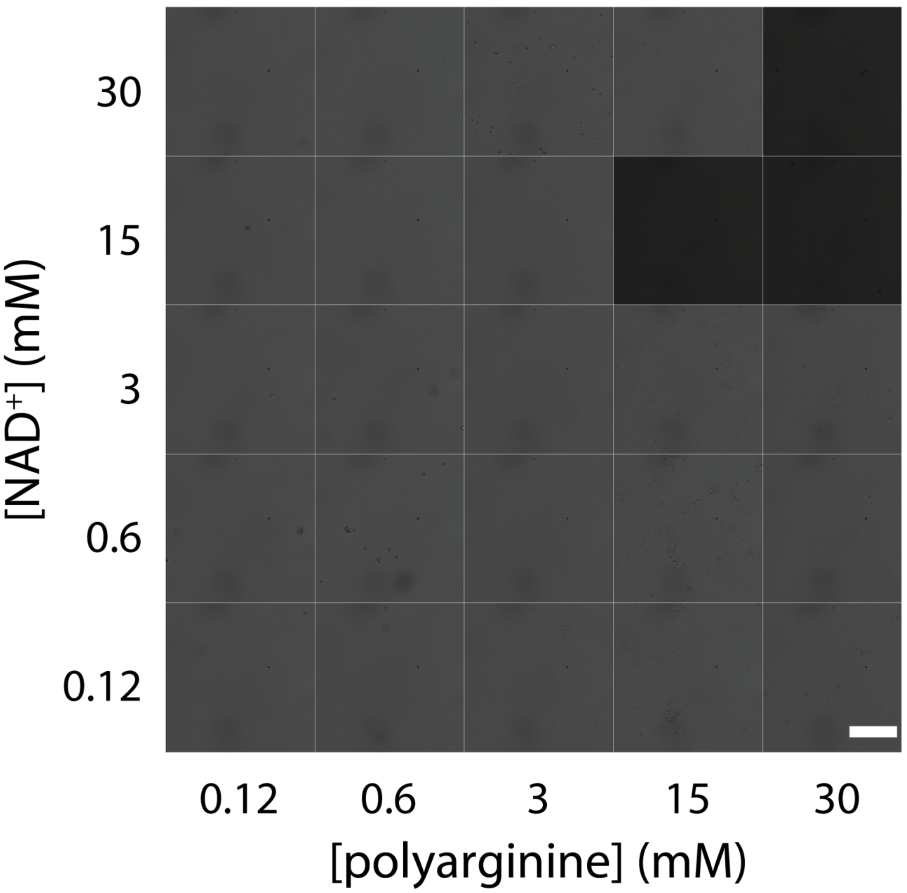

Figure S13. Bright field microscopy images mixtures of NAD^+ and 50-mer polyarginine, in 75 mM sodium bicarbonate, showing their phase behaviour, comprising of either dissolved, coacervated or precipitated state. To aid visualisation, carboxylate microspheres were added to samples that did not contain precipitates or coacervates. Scale bar : 20 µm.

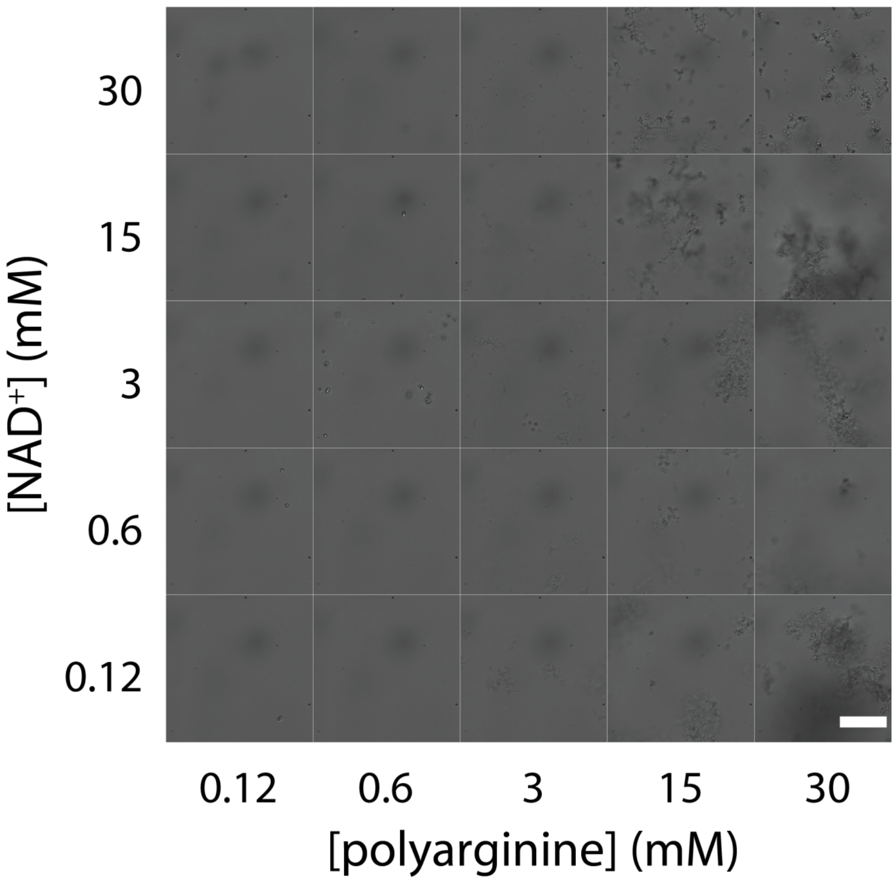

Figure S14. Bright field microscopy images showing mixtures of NAD^+ and 50-mer polyarginine, in 200 mM sodium bicarbonate, showing their phase behaviour, comprising of either dissolved or precipitated state. To aid visualisation, carboxylate microspheres were added to samples that did not contain precipitates. Scale bar : 20 μm.

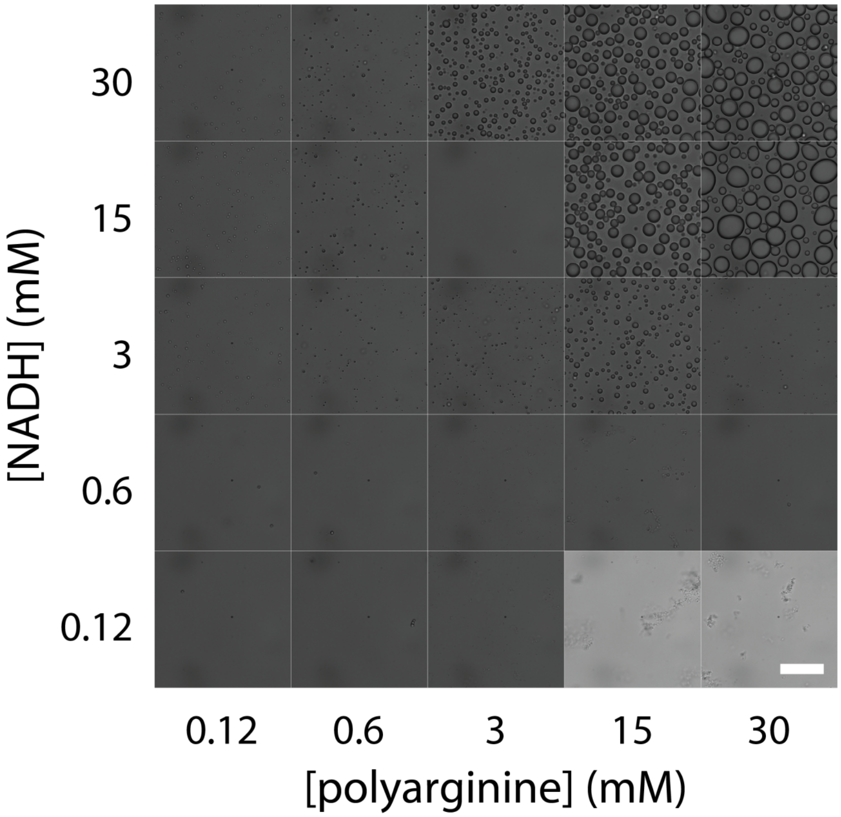

Figure S15. Bright field microscopy images of mixtures of NADH and 50-mer polyarginine, in 75 mM sodium bicarbonate, show regions of homogeneity, droplets and precipitates. To aid visualisation, carboxylate microspheres were added to samples that did not contain precipitates or coacervates. Scale bar : 20 μm.

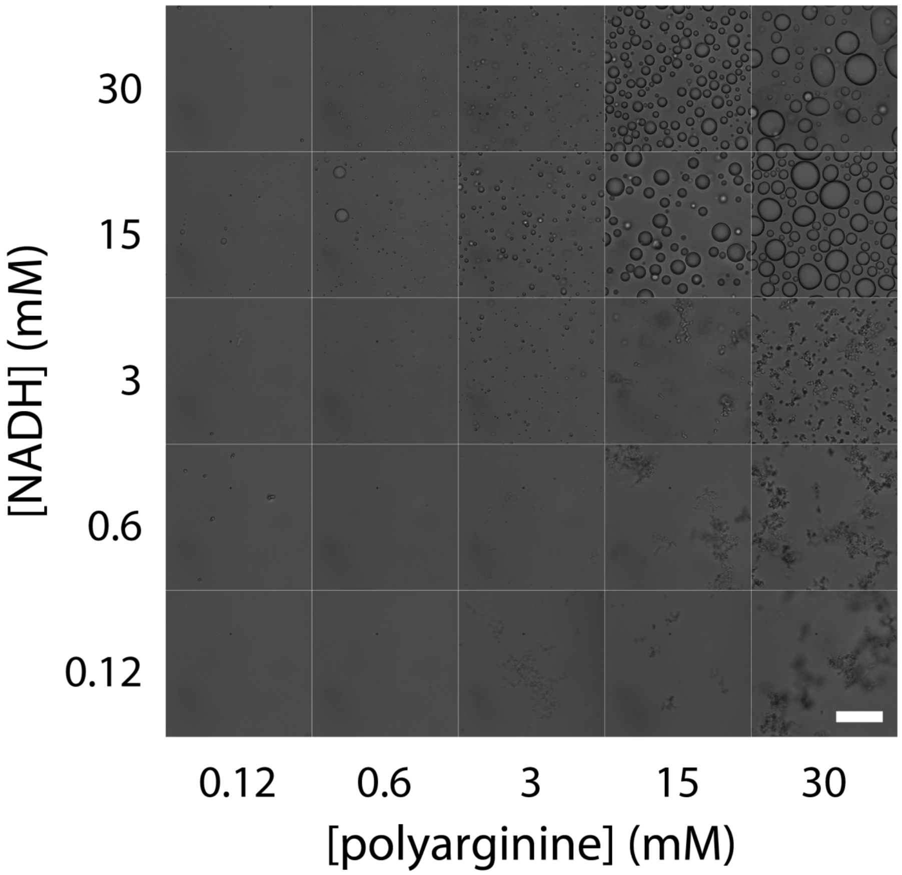

Figure S16. Bright field microscopy images mixtures of NADH and 50-mer polyarginine, in 200 mM sodium bicarbonate, showing their phase behaviour, comprising of either dissolved, coacervated or precipitated state. To aid visualisation, carboxylate microspheres were added to samples that did not contain precipitates or coacervates. Scale bar : 20 µm.

2.3. Supplementary note 1: Droplet formation is kinetically driven

To determine whether the phase transition from precipitates to droplets, driven by a protometabolic reaction, was a thermodynamically or kineticly driven process we loaded a dispersion of polyarginine precipitates comprised of 19.5 mM polyarginine and 200 mM sodium bicarbonate (pH 9) into an ibidi 18 Well µ-Slide adhered to a glass slide (see materials and methods). This was

placed onto a Nikon ECLIPSE Ti2 inverted microscope equipped with a AX-Galvo/ Resonant scanner and controlled via NIS -ELEMENTS software. After focusing the image with 20x CFI Plan Apochromat Lambda D lens, NADH was added to the ibidi 18 Well μ-Slide from one side to achieve a dispersion consisting of NADH (30 mM), polyarginine (15 mM) and sodium bicarbonate (200 mM) pH9. The sample was imaged every 5 seconds for 600 seconds with a PCO Panda 4.2 BI camera in wide-field mode.

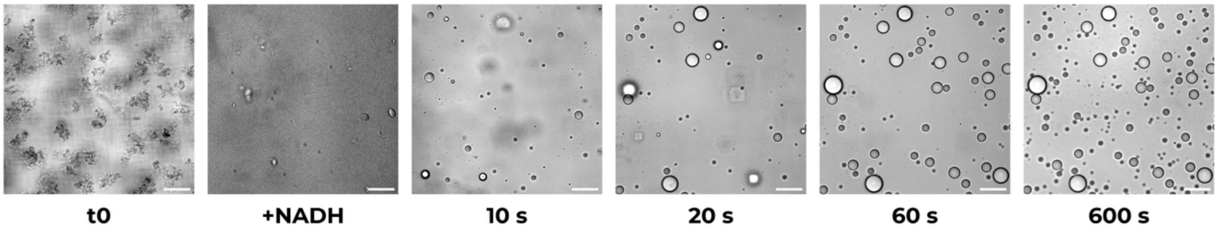

Figure S17: Widefield microscopy images show the transition from precipitates to droplets after the addition of NADH. Images show precipitates at t0 (the initial time point). Microscopy images taken immediately after the addition of NADH (+NADH) and at 10 s, 20 s, 60 s and 600 s show the formation of droplets. Comparing the timeframe for the precipitate to droplet transition after the addition of NADH (s) versus phase transitions induced by in-situ formation of NADH (min) indicates that the latter process is limited by the rate of NADH formation. Scale bar : 50 μm.

2.4. Determination of fraction of NADH produced

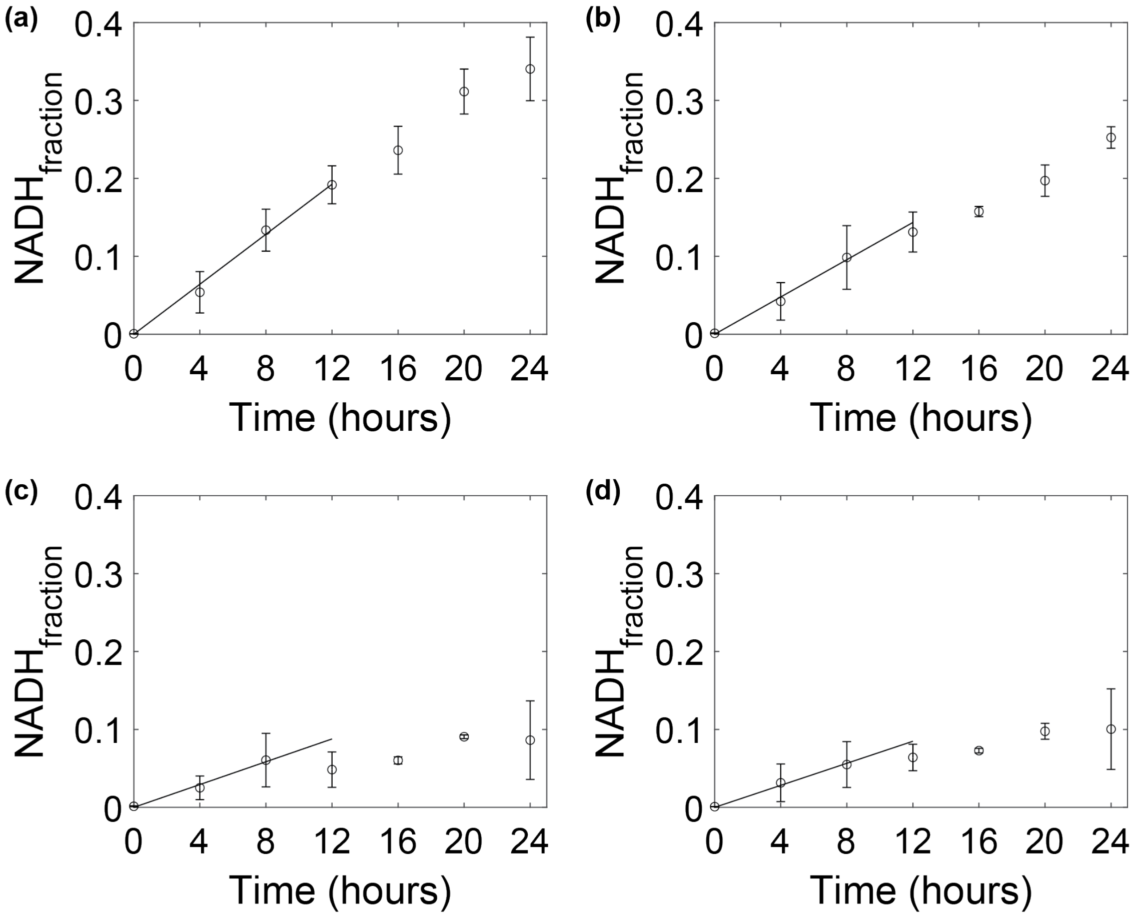

Figure S18. Linear least squares fit of NADH fraction to obtain the initial rate. Panels (a), (b), (c), and (d) show the NAD^+ reduction in the presence of 50-mer polyarginine in 75 mM sodium bicarbonate, 50-mer polyarginine in 200 mM sodium bicarbonate, only 75 mM sodium bicarbonate, and only 200 mM sodium bicarbonate, respectively. The slope of each fitted line indicates the initial reaction rate: 0.0160 ± 0.002, 0.0120 ± 0.0011, 0.0073 ± 0.0008, and 0.0071 ± 0.0006 hr^{-1}, respectively. The slope of fit has been extended from 8 to 12 hours for visualization. The fit comes from fitting to the first 8 hours. Error in the data points are the standard deviation from at least 3 repeats; the errors are calculated based on a 95% confidence interval.

2.5. Supplementary note 2: Multiphase behaviour of amphiphilic polyelectrolytes.

To test the ability for amphiphilic polyelectrolytes to exhibit multiphase behaviour, we tested the phase behaviour of chitosan (an amphiphilic polyelectrolyte) and poly(N-isoprophylacrylamide (PNIPAM) (an amphililic polymer) (Figure S19). For chitosan, a stock solution of 250 mM chitosan (monomer concentration) was prepared in 100 mM sodium acetate buffer at pH 5 or in PIPES buffer at pH 7.5. NADH was prepared as a separate 100 mM stock solution in buffer. Ammonium sulfate was prepared as a 1 M stock solution. For the final reaction mixture, the concentrations were adjusted to 15 mM Chitosan-HCl (monomer concentration), 100 mM buffer (either sodium acetate or PIPES), 30 mM NADH, and 60 mM ammonium sulfate by appropriate dilution of the stock solutions with matching buffer. Specifically, 50 μL samples were prepared by mixing 3 μL of 250 mM chitosan stock, 15 μL of 100 mM NADH stock, 3 μL of 1 M ammonium sulfate stock, and 29 μL of 100 mM buffer (sodium acetate pH 5 or PIPES pH 7.5).

For PNIPAM, a stock solution of 250 mM PNIPAM (molar charge concentration) was prepared in 500 mM carbonate buffer at pH 9. NADH was prepared as a separate 100 mM stock solution in buffer. For the final reaction mixture, the concentrations were adjusted to 30 mM PNIPAM, 500 mM carbonate buffer (pH 9), and 30 mM NADH. Specifically, 50 μL samples were prepared by mixing 6 μL of 250 mM PNIPAM stock, 15 μL of 100 mM NADH stock, and 29 μL of 500 mM carbonate buffer (pH 9).

In both instances samples were loaded into an ibidi 18 Well μ-Slide stuck onto pegylated glass slides and imaged using Nikon ECLIPSE Ti2 inverted microscope equipped with an AX-Galvo/ Resonant scanner and controlled via NIS -ELEMENTS software. Images were taken with a 20x CFI Plan Apochromat Lambda D lens and a PCO Panda 4.2 BI camera in wide field mode.

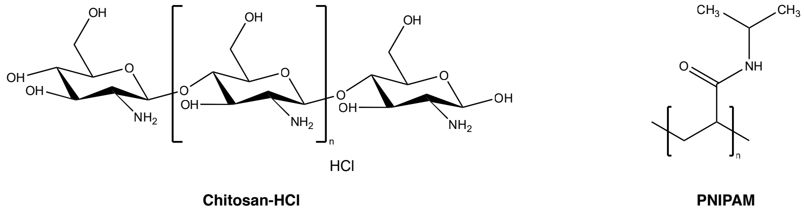

Figure S19: Chemical drawing for chitosan and PNIPAM

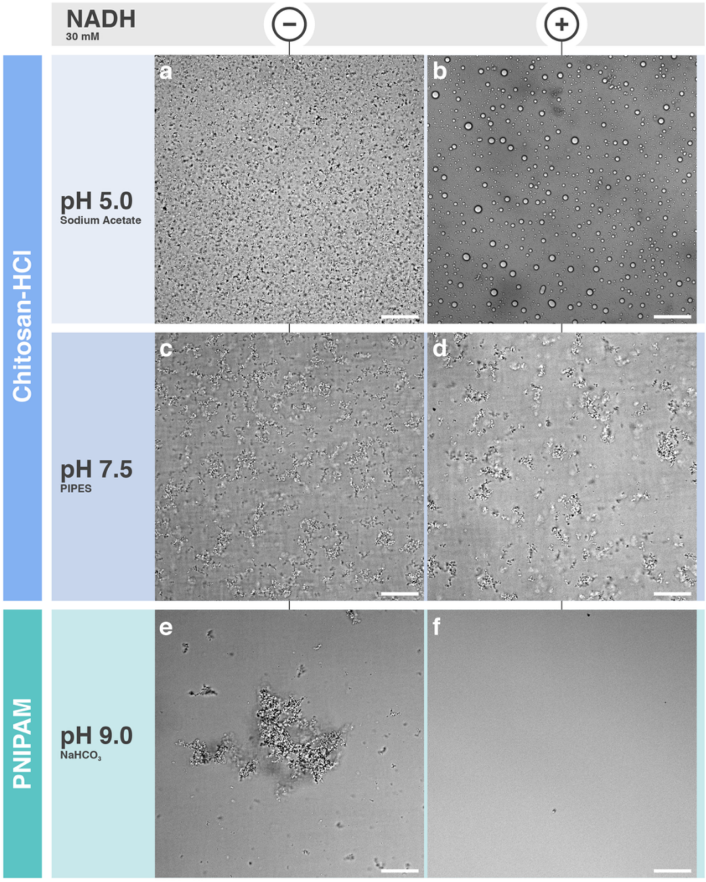

Figure S20: Wide field microscopy images of Chitosan and PNIPAM in buffer and in the absence and presence of NADH. Samples are chitosan (15 mM) in 100 mM sodium acetate buffer (pH 5) in the (a) absence of and in (b) the presence of NADH (30 mM) at pH 5.0 (top row) and in 100 mM of PIPES buffer in the (c) absence of and in the (d) presence of NADH (30 mM) at pH 7.5 (middle row). PNIPAM (30 mM) in 500 mM carbonate buffer (pH 9) in the (e) absence of and (f) presence of 30 mM NADH. Scale bar is 50 µm.

Our results show that at pH 5.0 chitosan will form precipitates with 60 mM ammonium sulphate in 100 mM of sodium acetate. Upon the addition of 30 mM NADH, droplets were observed. To test whether charge was important for tuning the phase behaviour of the polyelectrolyte we prepared an aqueous solution of chitosan at pH 7.5 above its pKa (6.5) to deprotonate the proton

on the primary amine. In the presence of 100 mM of PIPES buffer and ammonium sulphate at pH 7.5, chitosan forms a precipitate which remains a precipitate upon the addition of 30 mM NADH. This suggests that the hydrophobic moiety of chitosan is responsible for precipitate formation whilst the charge facilitates droplet formation. We next tested the phase behaviour of an amphiphilic, uncharged polymer (PNIPAAM). PNIPAAM formed a precipitate with 500 mM sodium bicarbonate and remained as a precipitate upon addition of NADH. Our results indicate that both hydrophobic and charged groups mediate complex phase behaviour. Under high salt conditions, precipitation is driven by the hydrophobic effect whilst the addition of charged molecules will induce a transition from a precipitate to droplets. These results indicate that mediation of the phase behaviour of hydrophobic polyelectrolytes by NADH could be a general phenomenon.

2.6. Supplementary note 3: Effect of metabolites on polyarginine-carbonate precipitates

To test effect of metabolites on precipitates of polyarginine in carbonate buffer we determined the ability for metabolites, ATP, CoA, FAD to induce phase transitions from precipitates to droplets. To do this, we prepared dispersions of polyarginine in carbonate buffer in the absence of and in the presence of ATP, CoA, FAD and NaCl at final concentrations of 75 mM for the carbonate buffer, and 30 mM for both polyarginine and small molecules. Dispersions were imaged in ibidi 18 Well µ-Slide stuck onto pegylated glass slides and imaged using Nikon ECLIPSE Ti2 inverted microscope equipped with a AX-Galvo/ Resonant scanner and controlled via NIS -ELEMENTS software. Images were taken with a 20x CFI Plan Apochromat Lambda D lens and a PCO Panda 4.2 BI camera in bright field mode.

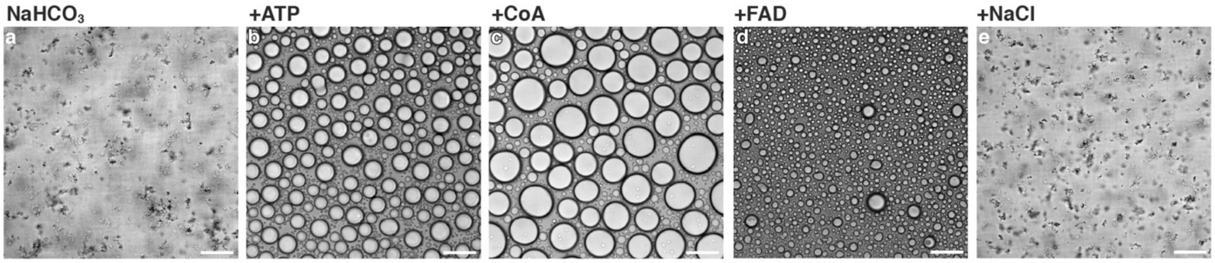

Figure S21: Widefield microscopy images of (a) polyarginine (30 mM) in carbonate buffer (75 mM) with 30 mM (b) ATP, (c) CoA, (d) FAD) and (e) NaCl. The results show that metabolites which are both amphiphilic and charged can induce a phase transition from precipitates to droplets in polyarginine-carbonate dispersions. The addition of NaCl at 30 mM shows precipitates indicating that charge alone is insufficient to induce droplet formation at equivalent concentrations to the metabolites. Scale bar is 50 μm.

2.7. Supplementary note 4: Effect of NADH on chicken egg white albumin

To test the effect of NAD^+ and NADH on chicken egg white medium we used a protocol as previously described[1]. Medium-sized, free-range chicken eggs were purchased from a supermarket and utilized before the stated expiration date. The egg white was separated from the yolk by careful shell-to-shell transfer. The chicken egg white was diluted 2-fold with 50 mM Tris/HCl buffer (50 mM, pH 7.4) and centrifuged at room temperature for 10 minutes at 5,000 × g. The supernatant was further diluted 5-fold with Tris/HCl buffer to a final concentration of 40 mM Tris-HCl. 40 μL of NAD^+, NADH prepared in TRIS-HCl buffer (50 mM, pH 7.4) were added to the 160 μL of supernatant to achieve a final concentration of 10 mM of NaCl, NAD^+ or NADH) and 42 mM Tris-HCl in a 0.2 mL thin-walled PCR tubes. The samples were incubated at 60°C in an Applied Biosystems™ MiniAmp™ Plus Thermocycler. As a control experiment, 40 μL of Tris-HCl was

added to the diluted chicken egg white albumin. Images were captured on a black background with a smartphone camera.

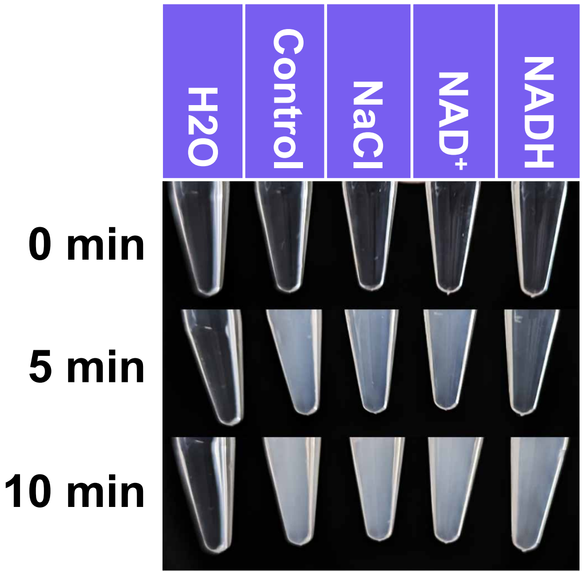

Figure S22. Images showing the increase in albumin turbidity with increasing time. Results show that NADH delays the onset of increased turbidity at 5 mins. 10 mM of NaCl, NAD^+, NADH or water were added to diluted chicken egg white to a final concentration of 10 mM for NaCl, NAD^+ and NADH respectively.

Supplementary references

1. Patel, A.; Malinovska, L.; Saha, S.; Wang, J.; Alberti, S.; Krishnan, Y.; Hyman, A. A. ATP as a Biological Hydrotrope. *Science* **2017**, *356* (6339), 753–756. https://doi.org/10.1126/science.aaf6846.
